# Empirical classification of fatigue-induced physiological tremor in robot-assisted manipulation tasks using BiLSTM-GRU network

**DOI:** 10.3389/fresc.2025.1474203

**Published:** 2025-06-17

**Authors:** Poongavanam Palani, Siddhant Panigrahi, Gunarajulu Renganathan, Yuichi Kurita, Asokan Thondiyath

**Affiliations:** ^1^ Robotics Laboratory, Department of Engineering Design, Indian Institute of Technology Madras, Chennai, India; ^2^ Biological System Laboratory, Cyber Physical System, Hiroshima University, Hiroshima, Japan

**Keywords:** muscle fatigue, physiological tremor, electromyography, mechanomyography, gated recurrent neural network, bidirectional long short-term memory

## Abstract

**Introduction:**

Physiological tremor arises due to stress, anxiety, fatigue, alcohol or caffeine. Under conventional circumstances, the physiological tremor would not be detrimental. Still, the mere presence of such a tremor during any microsurgical procedure can be catastrophic. In these instances, it is necessary to predict the progression of the tremor. This article proposes a novel sensing methodology and adds a distinctive feature to aid in classification. The classification of the progressive stages of fatigue-induced physiological tremor (FIPT) is based on the hybrid bidirectional long short-term memory neural network with a Gated Recurrent Unit (BiLSTM-GRU) presented in this work.

**Methodology:**

Twenty healthy participants volunteered in the study, where a teleoperation stage was set up using the Geomagic Haptic device—Touch. On the master end, the participants were seated comfortably and asked to trace the patterns embedded over an image of an organ that was displayed on the screen. The EMG and MMG_ACC_ signals from the Mindrove Armband and cross-sectional area changes, MMGCSAC, calculated from area measurement using the vision sensor, were recorded. The pattern-tracing task (PTT) was carried out over five repetitions, with fatigue-inducing exercise occurring between task epochs, thus accumulating fatigue throughout the data collection process. The extracted features from human movement aid the classification of the stages of tremor using BiLSTM-GRU, showing the significance of a cross-sectional area informed model.

**Results:**

The stages of progression of tremor are classified into five levels in this study, and classified using BiLSTM GRU with four different input feature sets. The performance evaluation metrics, such as the accuracy, precision, recall and F1 score, have been reported to ascertain the efficiency of the proposed feature group. The proposed feature set and classification strategy are capable of estimating stages of FIPT with 99% classification accuracy. This can be used to design state-of-the-art movement training platforms for both experienced and novice surgeons that allow informed decision making to attend to their tremor condition, either by taking a break or including a limb support to minimize its effects. At the same time, the identification methodology can be extended to pathological tremor rehabilitation and any other movement disorder diagnostics.

## Grapical Abstract

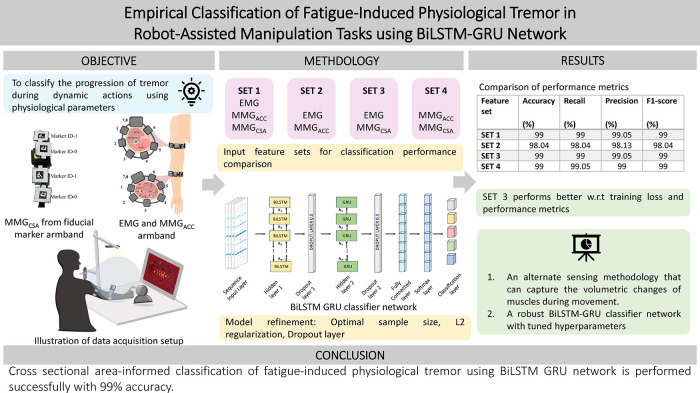

## 1 Introduction

Neurological conditions that affect human movement have been of interest for decades in sports, medicine, rehabilitation, and robotics. Some of these disorders require immediate rectification, while others require assistance and rehabilitation for an extended duration. The movement rectification strategy is needed when the person's movement disorder affects activities of daily living to a greater degree, or may affect task accuracy (1, 2). Tremor is the phenomenon where the body parts oscillate with undesirable amplitude and frequency, hindering regular movements. Based on the movement type, tremor can be classified into rest, postural, and action tremors based on the activity during which it occurs (3). During rest tremors, it can be noticed that the person has their body parts in a resting condition during which tremor occurs. Postural tremor occurs when the body part is held against gravity, while action tremor is found when some intentional activity is performed. Parkinson's tremor is a case where the high amplitude of the tremor causes inconvenience, and physiologic tremor is the form of induced tremor without pathological origins, which may be detrimental in some cases (4, 5). The physiological tremor is enhanced by fatigue, stress, anxiety, caffeine, alcohol or drugs, but is a reversible phenomenon (6, 7).

In robot-assisted manipulation systems, the human interaction with the robot makes the resultant motion unpredictable in cases involving hand tremor, affecting its application. This interaction needs to be studied to make conclusive decisions on improving the controllability of robotic platforms in collaborative environments. Robot-assisted micromanipulation has become increasingly crucial in diverse applications like laparoscopic surgery, micro-manufacturing, and micro-electronics assembly, extending to even nanoscale-level movements (8). In robotics-assisted surgery, the occurrence of cognitive and physical fatigue during long hours of surgery may adversely affect the outcomes. These tasks require precision and stability beyond the capabilities of a human operator. However, even with the assistance of robots, human hands are involved in supervising and controlling these robots, where FIPT can compromise the accuracy of such micromanipulation tasks. Assessment of surgeon fatigue is necessary to provide all-around support to surgeons and patients, avoiding any adverse events. In such scenarios, quantification of tremor is a primary step that can aid in identifying the onset of tremor for implementation of corrective actions, minimize work-related injuries and help in evaluating the levels of inaccuracy due to fatigue, which is pertinent for skills training in activities like suturing, teleoperation, and micro-pipetting. This necessitates the need to study the tremor level progression at the muscle level to provide key insights in suppressing it.

An attempt at quantification and evaluation of FIPT requires a basic understanding of the neurophysiological conditions that may affect the execution of smooth motion. The type of tremor can be characterised based on the frequency characteristics of the muscle activity or by estimating the external motion. The frequency of fatigue-induced physiological tremor is 8-14 Hz, and the indicator for the type of tremor is the increase in the root mean square amplitude of the signal and the decrease in the median frequency (9, 10). The measurement and characterization of physiological tremor has been carried out by many researchers and the same is discussed in the following sections shedding light on the various sensing methodologies and machine learning models that were built to serve the purpose.

### 1.1 Related research

Existing studies (11, 12) demonstrate that the amplitude of oscillations can be used to study both pathological (associated with high amplitude and low frequency) and physiological tremor (characterized by high frequency and low amplitude). The amplitude of oscillations can be a distinctive feature in identifying the progression of pathological tremors due to Parkinson's disease, Cerebral damage, or Diabetes Mellitus (13). Considering the trend of the amplitude of physiological tremors, a similar increase is also observed due to muscle fatigue, which can further induce a change in muscle contraction dynamics (14). Tremor frequency, on the other hand, can categorize whether the body part is at rest or moving. Micromanipulation focuses primarily on physiological tremor, which can be further classified into rest, postural, and kinetic tremors. Based on the frequency of motion, these oscillations can be distinguished into rest tremor (where no voluntary action is performed: 3–6 Hz), postural tremor (where the body part is held at a static pose against gravity: 4–12 Hz) and kinetic tremor (where particular target specific activity is performed to induce tremor: 3–10 Hz). This section discusses the multiple techniques used to characterize tremor and highlights the challenges of dimensionality reduction, technological complexity, and interpretability of such results.

Empirical classification and quantification of tremor require reliable measurement techniques. Several methodologies have been employed to quantify tremor, including wearable devices like accelerometers, gyroscopes, and electromyography (EMG) sensors. Accelerometers and gyroscopes provide physical motion characteristics, i.e., displacement, velocity, and acceleration, allowing researchers to analyze the frequency and amplitude of tremor (15). EMG measures electrical activity in muscles, giving insights into muscle fatigue. However, wearable sensors can be challenging to access indoors, leading to the development of optical trackers or pose measurement techniques to collect this data, trained on supervised learning human models (16). These techniques have enabled researchers to collect empirical data and classify fatigue-induced tremors based on their characteristics.

These quantitative measurements can study tremor features, but cannot provide a more subjective assessment of the tremor type, which requires learning of specific features independent of the movement or the body extremity involved. Since wearable/non-wearable sensors provide high-frequency data measurements that are voluminous, ever-changing, and user-dependent, machine learning algorithms can be effectively used to learn features from such complex datasets. A prerequisite step for any such learning algorithm is to extract useful regions of the data and summarize the entire data into a few training features, known as feature extraction. After extracting a few sets of parameters, they can be fed into the machine learning models. Machine learning models extract different features from the frequency and time domain of collected data (power distribution, median frequency, frequency dispersion) to differentiate typical human activity from tremor and characterize their source based on the activity performed.

Multiple studies provide evidence showing applications of machine learning algorithms to identify tremor and characterize their origin based on time and frequency-dependent parameters (18). Kostikis N et al. applied a tree-bagged classifier method to process a smartphone's accelerometer and gyroscope signals for remote classification of Parkinson's patients (18). In order to filter sensor noise and reduce data dimensionality, Jeon et al. implemented a support vector machine (SVM) for feature extraction and objectively analyzed the data using k-means nearest neighbour to cluster Parkinson's tremor severity (19). Demonstrated an intelligent joystick user interface that utilized SVM for feature extraction and a heterogeneous ensemble-based voting classifier capable of classifying activities of daily living from fatigue states with a detection accuracy of 92%. Probabilistic models like Markovian models were also utilized to measure the gait features before and after exhaustion, and such models were capable of detecting the onset of the fatigued stage with an RMSE of 0.83 ± 0.43.

Despite the potential of learning models in classifying tremors, segmenting tremor into multiple stages is not reported because of the requirement for large human subject data and a lower learning rate. Hence, this paper presents the methodology for the empirical classification of FIPT using recurrent neural networks and summarizes the performance of classification based on the feature sets utilized. This allows the user to decide on the ways to address FIPT based on its levels.

### 1.2 Potential contribution

The potential contributions of this work are:

1. Novel pipeline for evaluation of physiological tremor parameters and progression of tremor during dynamic actions. The transition into tremor must be identified to encounter the undesirable effects that can become detrimental in some cases. Hence, this paper proposes a BiLSTM-GRU neural network to aid in the classification of the stages of tremor progression.
2. New sensing methodology that measures cross-sectional area (CSA) from the volumetric changes of muscles during limb movement. An evaluation of the CSA-informed tremor classification is performed by feeding different combinations of the features to the network and evaluating the performance metrics.

## 2 Materials and methods

**Figure 1:**
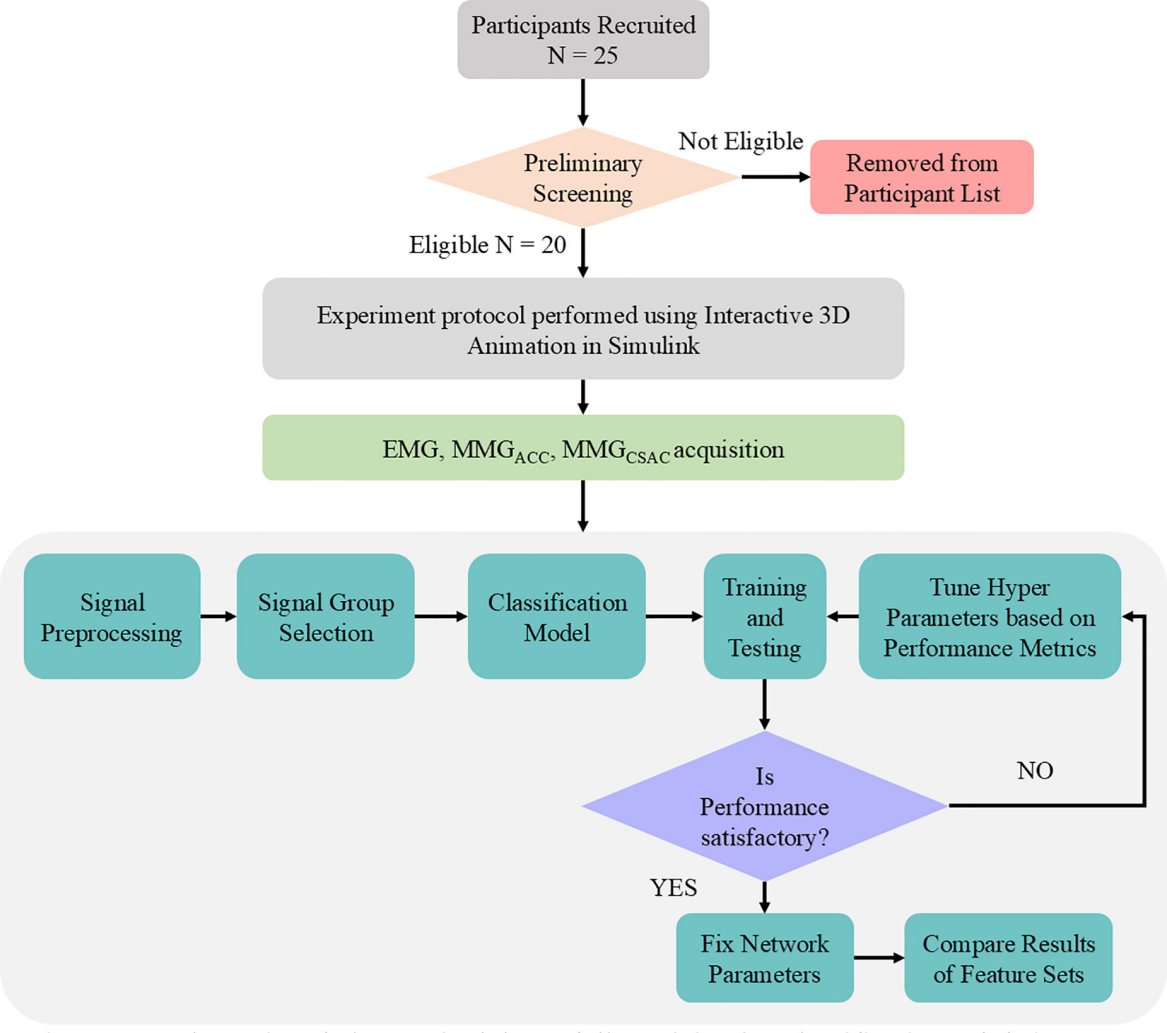
Schematic of the methodology followed in the classification of fatigue-induced physiological tremor progression for comparing the CSA-informed classification against the existing approach.

This work attempts to identify the levels of progression of FIPT by following the methodology as illustrated in Figure 1. The data is acquired from human participants who are made to perform progressive fatigue-inducing tasks. The EMG and MMG signals acquired during the process are cleaned by filtering and smoothing, and the process is explained in detail in Section 2.4, followed by labelling the data as per the class to which it belongs. In the “Level 1” stage (“No Tremor” stage), the participant does not have any tremor and is well-rested. In the “No Tremor” stage, the activities performed will not show any characteristics of fatigue or tremor. In the transition stage, “Level 2”, it is found that the participant begins to endure minimal fatigue. From the “Level 3”, the participants enter the fatigue stage. This is an important class to consider while evaluating the performance of the neural networks. In “Level 4” and “Level 5”, fatigue increases, manifesting in FIPT. These levels are classified based on the verbal inputs received from the participants during data collection to train the neural network.

Twenty healthy participants were recruited based on the inclusion criteria, such as the dominant hand being the right hand. The exclusion criteria for the participants are the presence of any movement disorders and a history of upper limb injuries. The participants were recruited based on social media advertisements within the Indian Institute of Technology, Madras, and the participant demographics are as given in Table 1. The signals acquired from human participants are electromyogram (EMG), accelerometer (MMG_ACC_) and cross-sectional area changes (MMG_CSAC_). The sensors used to acquire these signals are explained in Section 2.1, and the protocol implemented for data collection is discussed in Section 2.2.

**Table 1:** Participant demographics.

Variable	Mean	SD
Age (years)	28.2	3.188796
Height (cm)	165.65	11.07997
Weight (kg)	72.1	14.08956
Length		
Upper arm (cm)	32.625	2.901338
Lower arm (cm)	27.575	3.001206
Hand (cm)	17.885	1.553019

### 2.1 Sensors

#### 2.1.1 EMG and accelerometer armband

**Figure 2:**
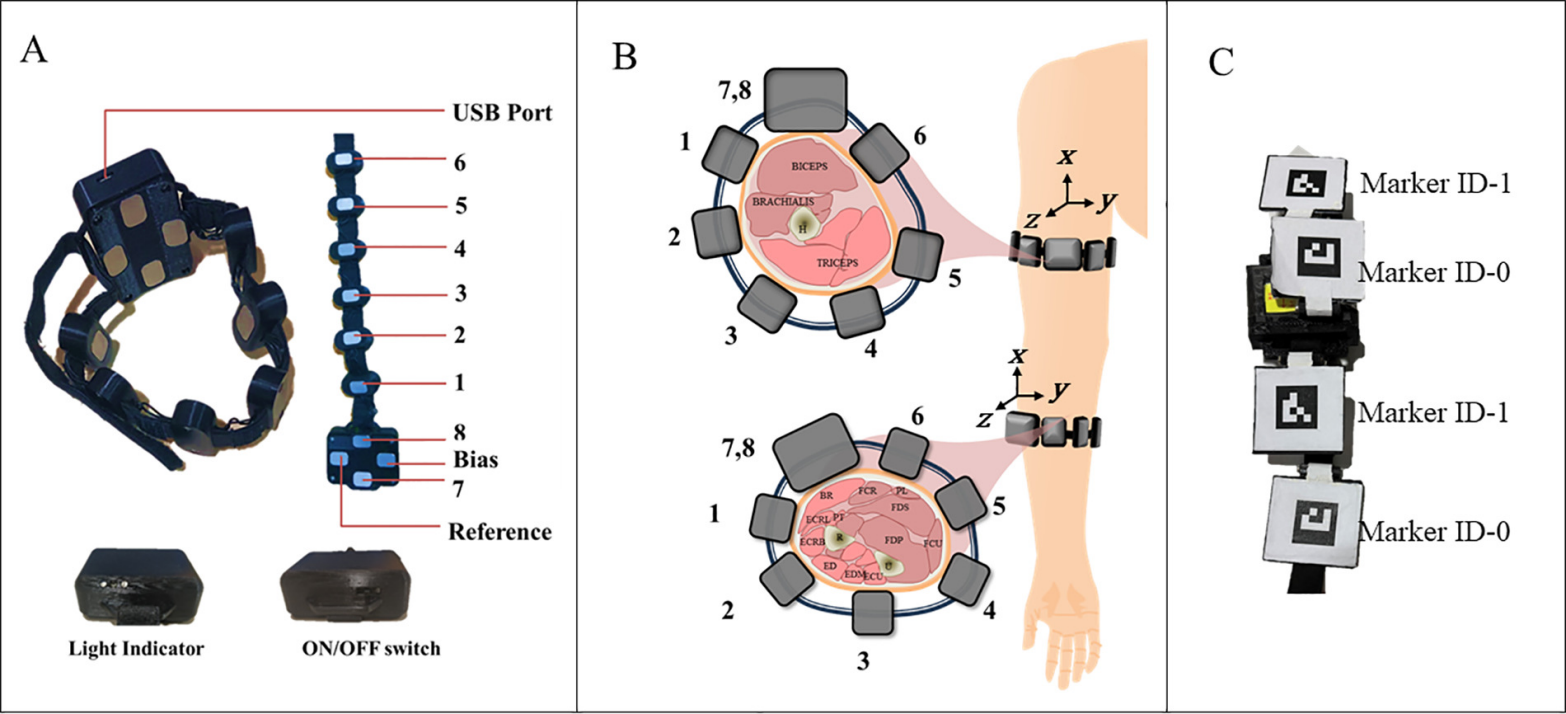
**(A)** Mindrove armband with parts labelled, **(B)** illustration of the muscles of interest and the sensor placement (FCU, flexor carpi ulnaris; FDS, flexor digitorum superficialis; ECU, extensor carpi ulnaris; ECRB, extensor carpi radialis brevis; ECRL, extensor carpi radialis longus; BR, brachioradialis), and C. Modified EMG band with fiducial markers.

Eight channels of EMG, along with accelerometer recordings, were collected from the forearm and upper arm muscles of the dominant hand of each human participant using the Mindrove EMG armband (Mindrove Kft, Budapest, Hungary) as shown in Figure 2A. The custom software Visualizer 2.3.2 was used to acquire and record the data at a sampling frequency of 500 Hz.

The muscles of interest in the upper arm are Biceps brachii, Triceps, and Brachialis, while the forearm muscles are Flexor Carpi Ulnaris (FCU), Flexor Digitorum Superficialis (FDS), Extensor Carpi Ulnaris (ECU), Extensor Carpi Radialis Brevis (ECRB), Extensor Carpi Radialis Longus (ECRL), and Brachioradialis. These are the muscles at the superficial layer and in contact with the EMG electrodes. The contribution of the extensor and flexor muscles during tremor plays a vital role (20), and this acquired using the 8-channel configuration as shown in Figure 2B. The MMG_ACC_ is present along with the EMG armband and is placed on top of the Biceps Brachii at the upper arm and the Brachioradialis muscle for the forearm. The cross-sectional area changes were monitored using an externally modified EMG armband as shown in Figure 2C, which will be discussed in detail in the subsection below.

#### 2.1.2 Cross-sectional area change measurement using modified EMG armband

The cross-sectional area changes were studied using a modified EMG armband with fiducial markers to measure them. Fiducial markers are employed in computer vision to identify objects, ascertain their spatial position as well as orientation, and reconstruct motion (21, 22). Despite their widespread utilization in odometry and navigation for mobile robotics, their application for measuring physiological changes while undergoing rehabilitation exercises is rarely explored. Visual-based fiducial markers, particularly ArUco markers, offer a portable and precise method for estimating changes in physiological cross-sectional area or girth resulting from muscular deformation. In contrast to flex sensors used for manual girth measurement, which restrict the range of motion and may impede precise microsurgical tasks prone to inducing tremors, this research focuses on exploring marker-based techniques. Traditional imaging methods, such as ultrasound and magnetic resonance imaging (MRI), are susceptible to background interference and are typically confined to clinical settings. Fiducial marking techniques, on the other hand, present a viable alternative for measuring forearm girth and its correlation with tremor progression. By strategically placing markers along the muscle belly, fiducial markers can be tracked in real-time, enabling the measurement of specific muscular segments for studying tremor progression.

**Figure 3:**
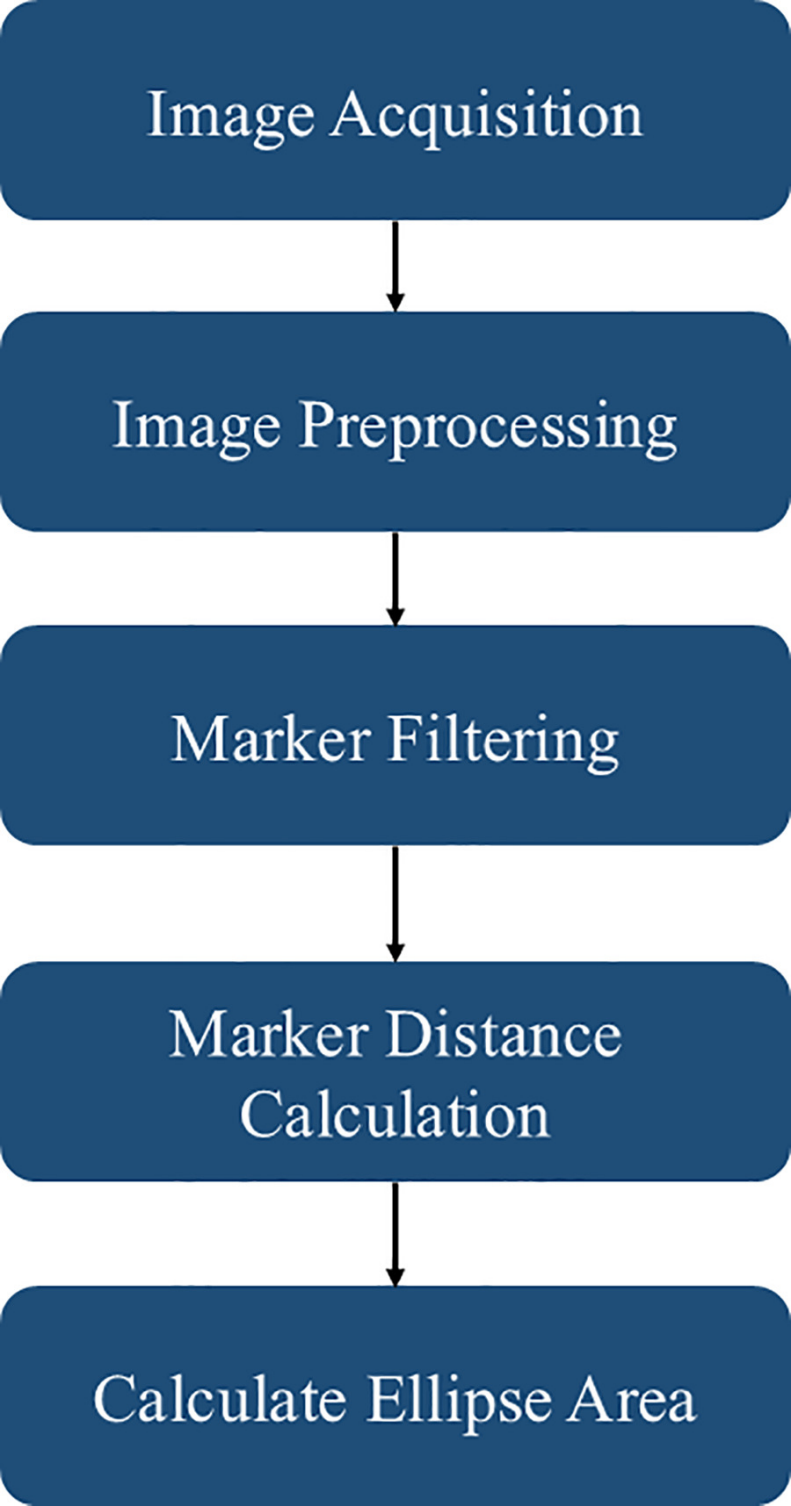
Proposed methodology for real-time cross-sectional area measurement.

The proposed setup uses two Lenovo 300 FHD cameras (2 MP CMOS sensor with DFOV 95°) to synchronize the ArUco markers in orthogonal directions. The input to the system is a stream of images from the two calibrated cameras. A 6 × 9 grid is used to calibrate the cameras with a spacing of 25 cm between the corresponding grids. After obtaining the input stream of data and the intrinsic properties of the camera distortion coefficients and principal point, the images are preprocessed to optimize the visual properties, thereby facilitating enhanced fiducial marker detection. Two markers, specifically, Marker ID-0 in the top view and Marker ID-1 in the side view, are detected. Following the detection of markers, the Euclidean distance between the centroids of the markers is calculated. The distances are also processed in pixels in the processed image of 640 × 480 pixels. Consequently, the known ArUco edge length (25 mm) converts pixel distances to centimeters. The resultant lengths from orthogonal perspectives are construed as an ellipse's major and minor axes, with the ellipsoidal area as an approximation for the cross-sectional area. The proposed methodology is represented as a flowchart in Figure 3.

**Figure 4:**
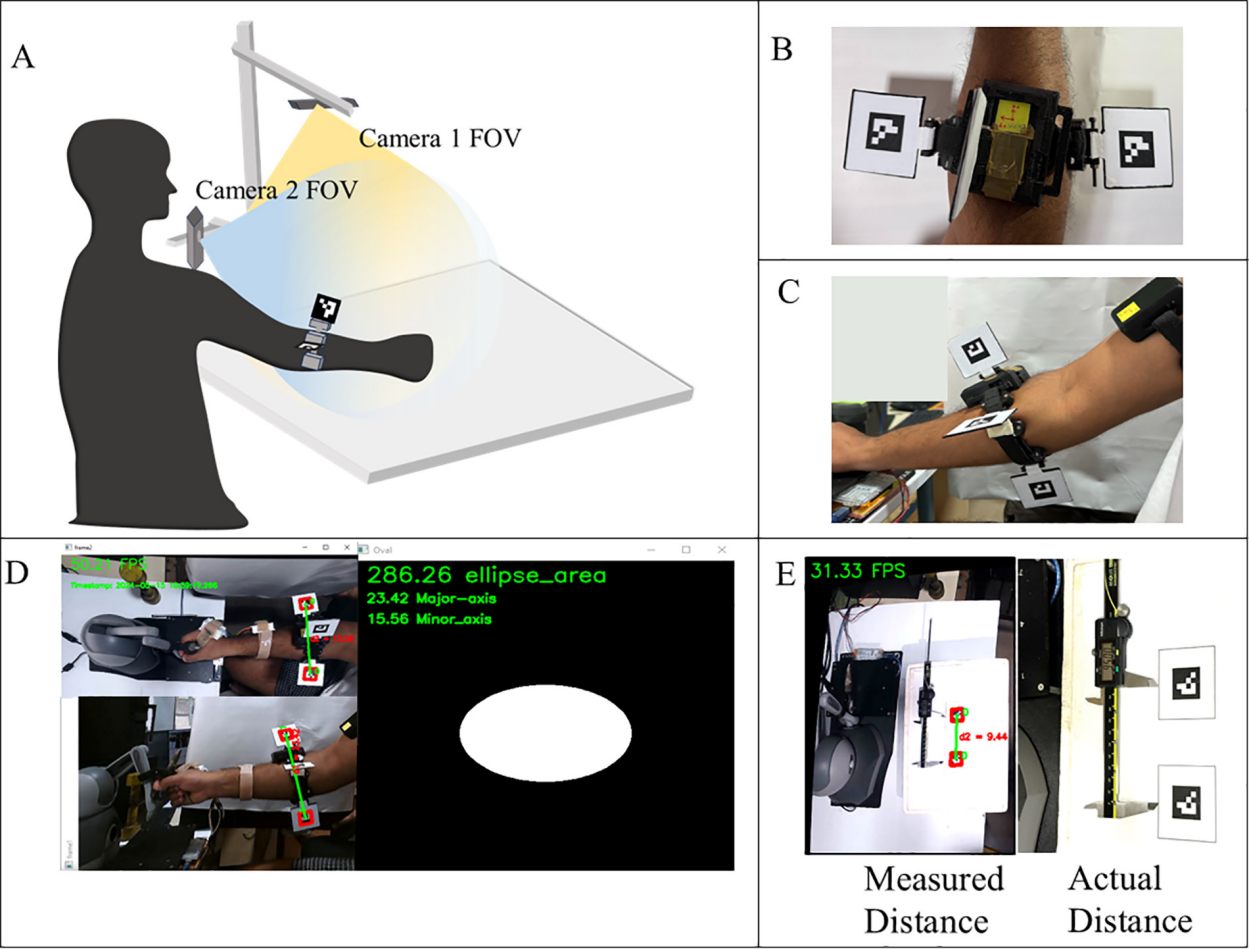
**(A)** Experimental setup of muscular girth measurement using fiducial marker, **(B)** top camera view of the fiducial markers, **(C)** Side camera view of the fiducial markers, **(D)** real-time display of fiducial marker system distance measurement with output for forearm girth measurement methodology proposed in this study, **(E)** evaluation of the accuracy of the proposed setup.

The experimental setup and the orthogonal placement of a pair of ArUco markers for cross-sectional area estimation are illustrated in Figure 4. Figure 4A shows the real-time cross-sectional area acquisition setup, and Figures 4B,C depict the exploded view of the custom-made hinges with bi-coloured ArUco markers fixed to the Mindrove EMG armband in orthogonal directions to measure the cross-sectional area of the forearm.

These alterations in physiological cross-sectional areas due to manipulation tasks performed by the participant can be quantified by monitoring changes in major and minor axis lengths, achievable through the proposed setup. Figure 4D illustrates the system output of the forearm on cross-sectional area estimation using computer vision. The output from the first camera (top view) and the second camera (side camera) is used for the area measurement by approximating the forearm to an ellipsoid with the given major and minor axis lengths.

The accuracy of the setup for its study in biomechanical studies is performed by measuring known distances. This procedure is repeated for five trials for both the orthogonal cameras, and it is observed that both the cameras can measure distance with a mean average error of ±0.1 mm as shown in Figure 4E. The proposed measurement methodology can detect the ArUco markers with a mean averaged error of 6.81% and repeatability of 0.117 mm. Extensive experimentation has demonstrated the robustness of the setup for studying tremor progression; however, certain limitations remain. High-frequency motion and the size of fiducial markers can impact marker detection. Occlusion and indoor settings may also induce false positives. Intrinsic calibration was performed to determine the optimal distance (60 cm), marker length (25 mm), and frequency of motion (36 bpm metronome beat) to mitigate these limitations.

### 2.2 Experiment protocol and data collection

Ten healthy male and ten healthy female participants were recruited for the study based on social media advertisements. The participants provided written consent to volunteer for the study, approved by the Indian Institute of Technology Madras Ethics Board (IEC/2021-02/TA/09). The participant demographics are listed in Table 1, along with the upper and forearm girth measurements. They were given time to get accustomed to handling and moving the haptic device in the environment created using Simulink 3D Animation. They were given a brief explanation regarding the experiment protocol and the patterns that will be part of the study.

**Figure 5:**
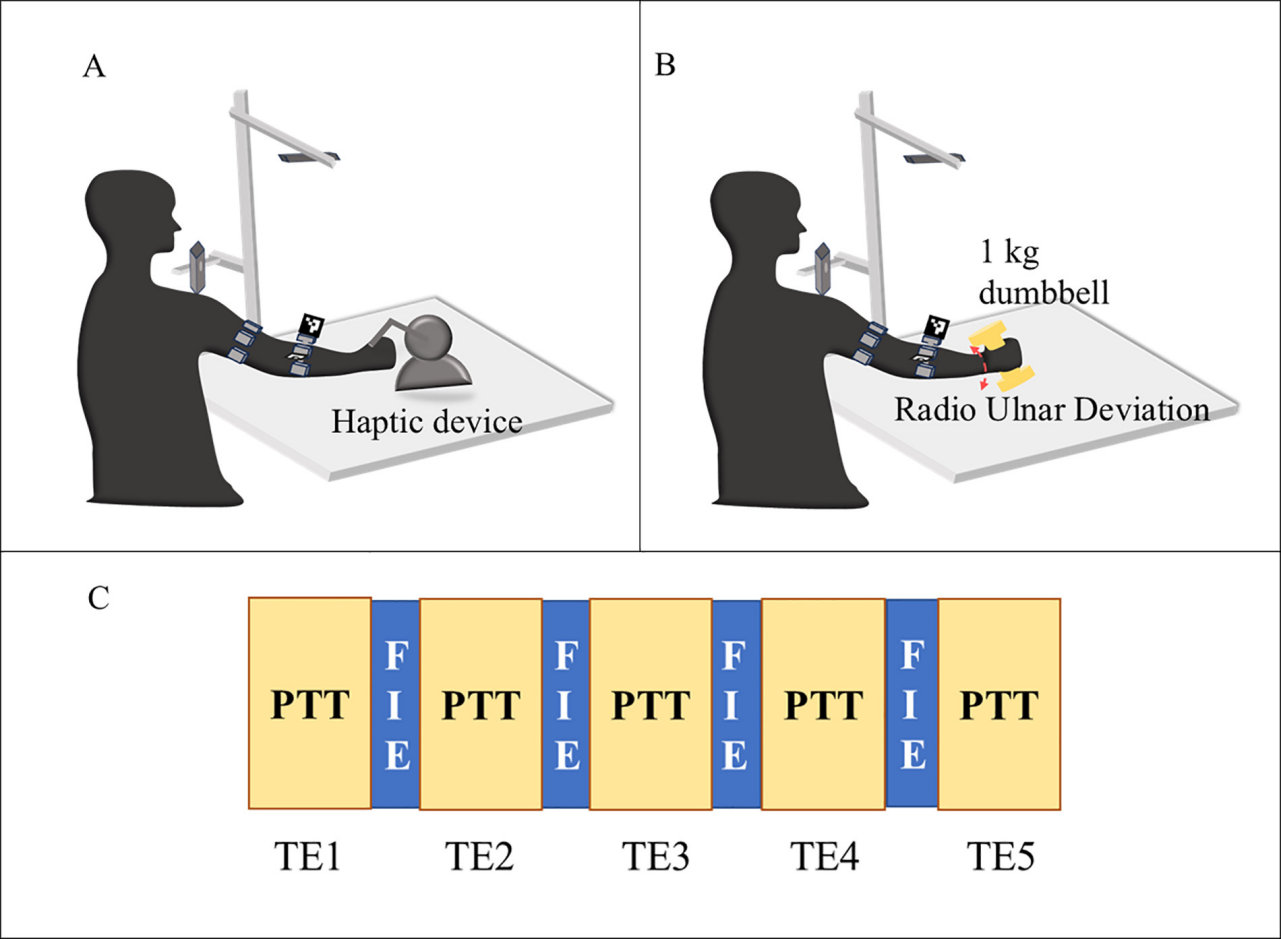
Illustration of the experiment protocol **(A)** pattern tracing task (PTT) **(B)** fatigue inducing exercise **(C)** flow of the task epochs (TE) with pattern tracing task (PTT) followed by fatigue inducing exercise (FIE) for 5-time.

**Figure 6:**
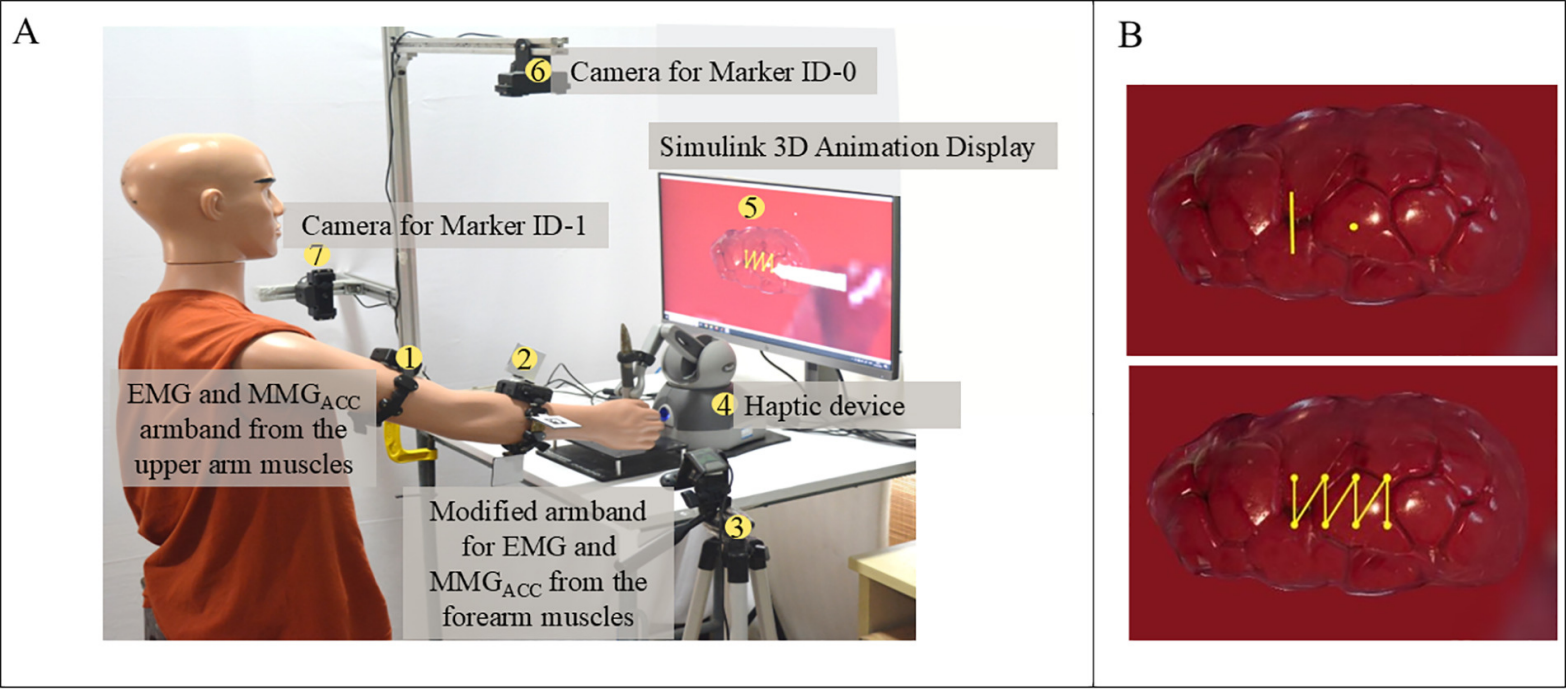
**(A)** Experimental setup for data collection using modified EMG armband and vision sensor, **(B)** patterns traced for the study static holding task (SHT) and dynamic line tracking task (DLT) (top) and interrupted suture task (IST) (bottom).

The participants were positioned comfortably in a chair without an armrest to avoid resting their arms between sessions, which can impede the development of arm fatigue, as depicted in Figure 6A. The protocol involves the participants tracing patterns repetitively with physician-approved fatigue-inducing tasks between repetitions. The protocol consists of the participant holding the end effector tool of the haptic device and tracing the pattern displayed on the screen five times for the first epoch, followed by a physician-approved fatigue-inducing exercise (FIE) for 40 s. The FIE involves the participant carrying a 1 kg dumbbell and performing radioulnar deviation about the wrist, holding the elbow away from the body without any elbow support. The participants were asked to perform sustained maximum contractions when reaching the maximum angle of flexion and extension as showin in Figure 5B. This combination of repeated fatigue-inducing contractions performed between the task epochs helps build muscle fatigue for the duration of the experiment. Then, the same pattern is traced five times for the second task epoch, followed by the fatigue-inducing exercise. A total of five such task epochs are performed for each pattern as seen in Figure 5C. The trials were time and speed-controlled using a metronome to ease the inter-subject signal analysis. To ensure that the participant is completely rested and muscles are not fatigued before they begin the next pattern set, an average of the 24 h interval is provided.

The data acquisition setup is as shown in Figure 6A with the participant wearing the armbands and tracing patterns using the haptic device. The haptic device (Geomagic Touch) is connected to the Simulink environment using a custom S-function block from Quarc 2020 SP2(4.0.3271) (Quanser Consulting Inc., Markham, Canada), which provides the haptic device encoder output. The end effector position is obtained by calculating the forward kinematics using Simulink blocks, and the output position is linked to the 3D Animation Visualizer sink. This connects the end effector tool of the haptic device to the “pen” visualized in the 3D gaming environment, as shown in Figure 6A. The participant holding the end effector tool (“pen”) of the haptic device will perceive haptic feedback on touching the kidney in the Simulink environment. The participant moves this pen to trace the pattern while being able to perceive the force feedback from the pen while touching the surface of the kidney (an organ representation in the interactive simulation environment).

The patterns, as illustrated in Figure 6B, have been designed to closely represent the manipulation in surgical motions. This was created using the Simulink 3D Animation blocks with a 3D view of a kidney with patterns to be traced embedded, thus providing a gaming environment with Unreal Engine. The patterns to be traced enforce movements that replicate simple suturing tasks, assessing the required dexterous motions. Figure 6B (top) has the static tool holding task where the participant is asked to hold the tool on the yellow dot for 20 s and then move to the “line” pattern where they will be asked to trace the yellow line up and down using the haptics end effector tool. For the second pattern set, as shown in Figure 6B (bottom), a zig-zag line replicating the interrupted suture is presented to the participant. The tracing begins from the right corner to the left corner. The signals recorded are to be processed before they can be introduced to the neural network. The following section explains the data preprocessing and feature engineering of the acquired signals. During the experiment, data were collected from the accelerometers, EMG sensors and cross-sectional area from vision sensors. The following section explains the data preprocessing and feature engineering for tremor level classification purposes.

### 2.3 Data processing and feature engineering

**Figure 7:**
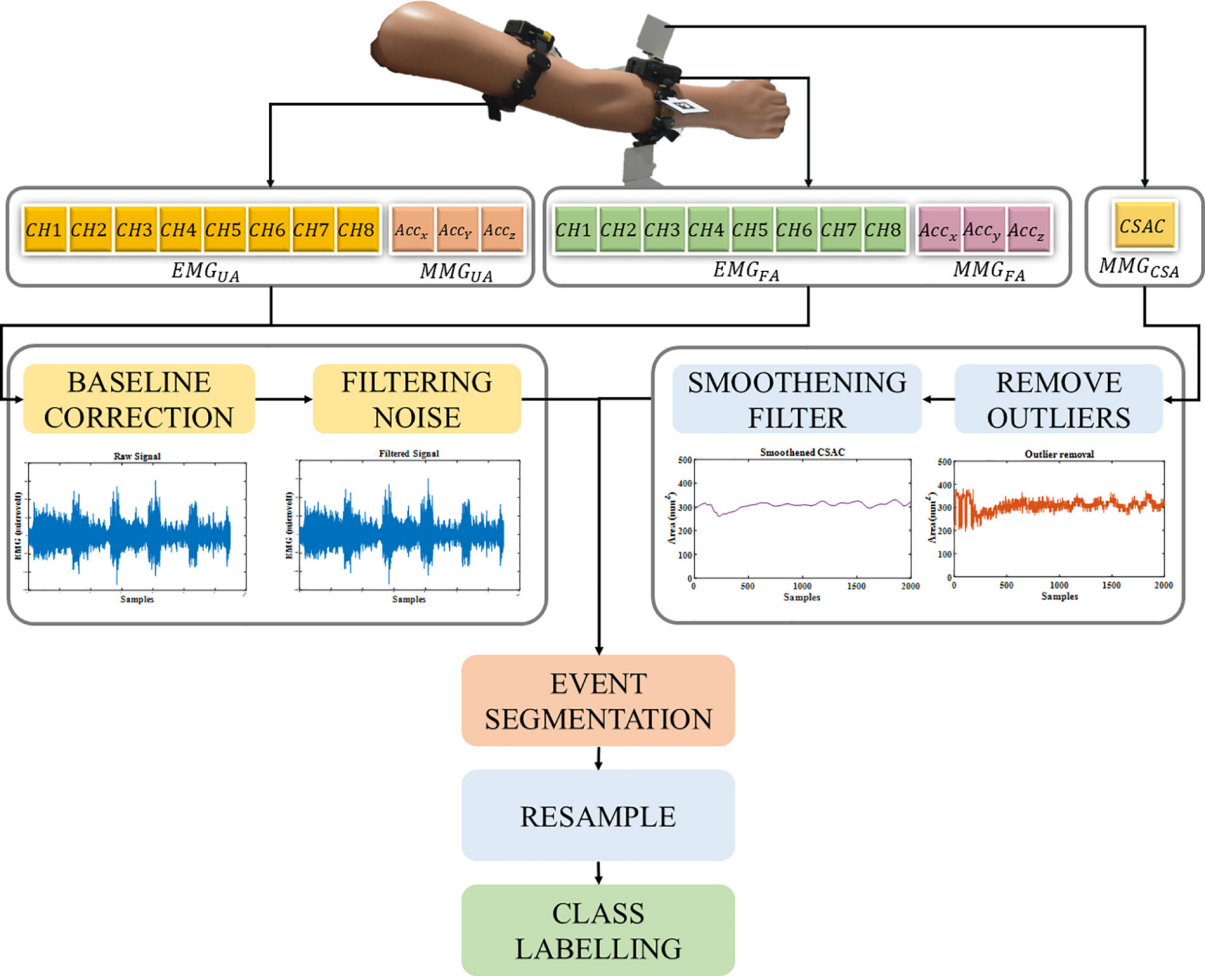
Signal preprocessing pipeline for feeding the classifier, depicting the signal acquisition using an EMG armband around the upper arm muscles, providing $\mathrm{EM}G_{\mathrm{UA}}$ and $\mathrm{MM}G_{\mathrm{UA}}$ and a modified EMG armband on the forearm arm muscles, providing $\mathrm{EM}G_{\mathrm{UA}}$, $\mathrm{MM}G_{\mathrm{UA}}\;$ and with $\mathrm{MM}G_{\mathrm{CSAC}}$ calculated from the vision sensor.

The preprocessing of all the signals is performed as shown in the block diagram (Figure 7) using MATLAB 2022a software. The raw data is cleaned, and the outliers are removed as part of feature engineering. The preprocessed signals are then segmented into the tremor stages, followed by time normalization over the multi-sensor signals, and task epoch-based class labels are created appropriately.

#### 2.3.1 Electromyogram (EMG) and accelerometer (MMG_ACC_)

The Mindrove armband is used to acquire EMG and accelerometer (MMG_ACC_) signal measurements as dimensionless numbers, which should be later processed to convert into µV for EMG signals and *g* value for the accelerometer. The DC offset correction is performed on the multichannel EMG signals and then filtered using a Butterworth bandstop filter of the second order to remove 50 Hz noise interference. This signal is further rectified, and the signal's envelope is created. For the MMG_ACC_, the root sum of squares of the x, y and z direction acceleration vectors is calculated as a time domain feature using equation (1).

(1) $$\mathrm{MM}G_{\mathrm{ACC}}=\sqrt{\mathrm{MM}G_{ACCx}^{2}+\mathrm{MM}G_{ACCy}^{2}+\;\mathrm{MM}G_{ACCz}^{2}}$$

#### 2.3.2 Cross-sectional area changes (MMG_CSAC_)

The cross-sectional area is estimated in real-time and recorded as explained in Section 2.1, and further processing is performed to remove outliers using a moving median filter. The filtered data is then smoothed using the Savitzky-Golay polynomial filter of the fifth order. This step is necessary to avoid environmental noise caused by clutter and/or scattered light in the captured frames.

(2) $$\begin{array}{c} MMG_{CSAC}\;(i)=\;\frac{Area_{i}-\;Area_{rest}}{Area_{rest}} \end{array}$$

where, *i* is the current frame. The relative change *MMG_CSAC_* is calculated against the resting condition $Area_{rest}$.

The features are resampled since EMG and MMG_ACC_ have sampling frequencies of 500 Hz, and the cross-sectional area measurements come from the 50 fps camera. The data acquisition system is different for the sensing modalities, hence the signals are resampled to 250 Hz. The signal is normalized to zero mean and unit variance before feeding it into the classifier network. The number of participants is 20, and the dataset to feed a classifier network should be large. Hence, the time series is augmented using the window slicing method with a desired window size of 1,000 and the number of windows set to 50, making the model more robust by introducing variability in the dataset (23, 24).

The time-frequency domain features were extracted using multivariate empirical mode decomposition and analysed using the Hilbert transform to identify the task epoch level distinction (25). The class labels are created based on the number of the task epoch, which will be five classes for the increasing levels of FIPT. The features that were given as input to the neural network are the eight channels of EMG and MMG_ACC_ from the upper arm and forearm, and the MMG_CSAC_ from the forearm.

The tremor classification studies performed in the literature involve either a classification between Parkinson's and essential tremor or between the presence and absence of tremor. The different classification networks and their performance metrics are listed in Table 2. Some of the neural networks used are the multilayer perceptron (MLP), Support Vector Machine (SVM) using radial basis function (RBF) kernel, and k-nearest neighbour (KNN). The performance metrics are compared, and it can be seen that the sequential data classifier LSTM performs better in terms of classification accuracy, and its ability to classify unbalanced datasets is also good based on its F1 score.

**Table 2:** Comparison of classification networks in the literature used for differentiating tremor.

Author & Year	Classification Network	Sensing modality	Classification categories	Accuracy	Precision	Recall	F1 score
(26)	MLP using Scaled-conjugate (SCG) learning algorithm	EMG	ET vs. PD vs. Normal	88	89	88	-
	MLP using Broyden–Fletcher–Goldfarb–Shanno gradient learning algorithm (BFGS)			91	87	92	–
(27)	SVM using RBF kernel	EMG, Acc	PD vs. ET	83	–	–	–
(28)	MLP	EMG, Acc		92.5	95	89.7	–
(29)	KNN	EMG	Tremor vs. No Tremor	84	95.5	68	79.8
	SVM using RBF kernel			90.5	89	91.5	85
	RF			84.5	84	96	84.5
	LSTM			97.5	97	98	98.5
	KNN	Kinematics		94	90	89	94.5
	SVM using RBF kernel			91	86	84	91
	RF			95.5	96	95.5	95.5
	LSTM			96	96	96	96

ET, essential tremor; PD, Parkinson's disease; Acc, accelerometer.

The recurrent neural network (RNN) is a supervised learning approach that can aid in sequential data classification due to its ability to store memory and make decisions based on this memory. The basic RNN consists of a tanh function and suffers from short-term memory. An advanced version of this is the Long Short-Term Memory (LSTM) network with a memory cell state containing gates to decide how much memory will be retained. In this paper, an enhanced version of the LSTM network is applied, and this is discussed in detail in the following section.

## 3 BiLSTM-GRU network architecture

A recurrent neural network allows prediction (30, 31) and classification (32) of time series data. The LSTM is a type of recurrent neural network that can perform selective reading, writing, and forgetting using a backpropagation loss function to make these selection decisions (33). This allows the LSTM to address the vanishing gradient problem of the recurrent neural network (34). The network contains “gates” to capture long-term and short-term memory in and out of the cells, thus aiding in the classification of fatigue-induced physiological tremor time series. The input gate collects the input features into the cell state, while the forget gate uses weighted parameters to decide whether it should retain or forget the information. The output gate is the third gate for the cell state output.

**Figure 8:**
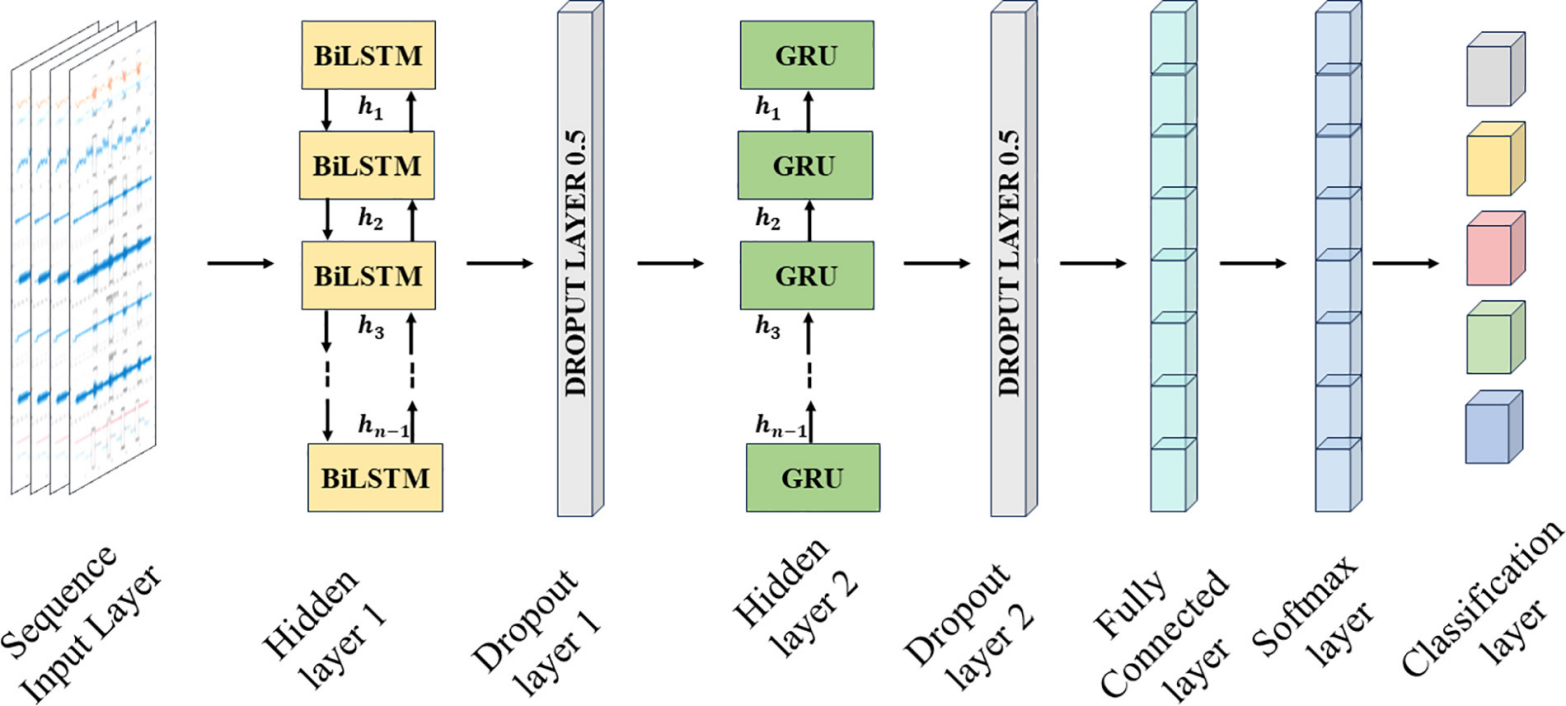
Architecture of BiLSTM-GRU neural network for three combinations of input feature groups.

A BiLSTM is a two-way architecture as shown in Figure 8, thus performing forward and backward state traversal, which increases the accuracy for long-time series (35, 36). The Gated Recurrent Unit is one step less than LSTM since it combines the input and forget gate into the update gate, thus reducing the number of parameters and increasing the speed of convergence. The input to the neural network is $x=\;(x_{1},x_{2},\ldots \;x_{T})\in R^{T}$ through the LSTM layer, where T=S×N with *S* as the number of segments to split the original time series of length *L*, and *N* is the number of features. So, the number of iterations would be $I\;=\;\frac{L}{B}\;$ where *B* is the batch size.

### 3.1 GRU layer

The chosen neural network is a GRU-based BiLSTM network owing to the fact that the classification can be performed on the interdependencies existing in the tremor time series data acquired. In this sequence-to-sequence classification, the number of features is in the columns of the input vector. The GRU layer is added to address the disadvantages of the LSTM neural network, which again contains 50 hidden units.

### 3.2 Softmax layer

The softmax layer in a multi-class classification neural network, such as the BiLSTM-GRU network, allocates the probability that the input belongs to one of the five classes. The activation function is generally deployed in the final layer of this network. The raw output is converted into probability scores using equation (3).

(3) $$\begin{array}{c} \mathrm{softmax}\;{\left(x\right)}_{i}=\;\frac{e^{x_{i}}}{\sum_{\;j=1}^{C}e^{x_{j}}} \end{array}$$

where, *x* is the raw output from the BiLSTM-GRU network, *i* is the current predicted class, and C is the total number of classes.

### 3.3 Classification layer

This layer usually comes after the SoftMax layer, and it computes the cross-entropy loss for *C* mutually exclusive classes using equation (4) (37). Based on the outputs from the SoftMax layer, the classification layer assigns each input sequence to one of the five classes. The five levels identify the progress from resting mode to levels of activity, with three sets of feature groups. The attempt is to identify if the proposed sensing methodology of features aids in better classification and how the classification accuracy varies for each feature group. Hence, there are five classes for any given set of features. The cross-entropy loss between the predictions and the targets is calculated as:

(4) $$\begin{array}{c} \mathrm{Cross}\;\;\mathrm{entropy}\;\mathrm{loss}=\;\frac{1}{L}\overset{L}{\underset{n=1}{\sum }}\overset{C}{\underset{m=1}{\sum }}w_{m}t_{nm}\ln y_{nm} \end{array}$$

where *L* is the number of samples, *C* is the number of classes, $w_{m}$ is the weight of the class *i*, $t_{nm}$ is an indicator that the signal belongs to the $n^{th}$ input belongs to *m* class and $y_{nm}$ the output of the softmax layer.

To avoid overfitting and enhance learning, dropout layers are introduced after the BiLSTM layer and the GRU layer that selectively removes a set of neurons from participating in the forward and backpropagation. Also, L2 regularization is performed to aid in achieving better classification accuracy by imposing a penalty on the loss function. The chosen optimizer is the Adam optimization technique. These aid in better learning for the classifier and faster convergence.

## 4 Results

The neural network was trained and tested in a system with a hardware configuration of CPU: Intel Core i90 10900, GPU: NVIDIA GeForce RTX 2060 SUPER and 16 GB DDR4 RAM. The BiLSTM -GRU neural network was built using MATLAB R2022a software with dedicated deep learning toolboxes installed. The training details and the layers employed are listed in Table 3. The BiLSTM layer contains 50 hidden units and the GRU layer contains 50 hidden units.

**Table 3:** Hyperparameters for the BiLSTM-GRU neural network.

Hyperparameter	Value
No. of hidden units in LSTM	50
No. of hidden units in the GRU layer	50
Dropout layer	0.5
Optimizer	Adam
L2 regularization	0.01
Mini Batch size	64
Maximum Epochs	30
Initial learning rate	0.001
Learn Rate Schedule	piecewise
Learn rate drop factor	0.1
Learn rate drop period	10
Gradient threshold	1
Execution environment	GPU

The zero-mean unit variance features are labelled and fed into the neural network. In this, the data is divided into ten parts: nine parts for training (training set) and one part for validating (test set) the neural network. The test set is then evaluated for performance metrics, and the comparison is discussed in the following sections. The network is trained using different combinations of the feature sets to evaluate the significance of the proposed sensing methodology. The feature sets are

• Feature Set 1—EMG, MMG_ACC_ and MMG_CSAC_
• Feature Set 2—EMG and MMG_ACC_
• Feature Set 3—EMG and MMG_CSAC_
• Feature Set 4—MMG_ACC_ and MMG_CSAC_

The EMG contains eight channels each for the upper arm and the lower arm, hence the time series obtained is sixteen. The MMG_ACC_ contains two time series of signal magnitude vector calculated over the x,y and z axes acceleration vector, each for the upper arm and lower arm. The MMG_CSAC_ contains one time series calculated for the forearm. Hence, feature set 1 contains 19 inputs, feature set 2 contains 18 inputs, feature set 3 contains 17 inputs, while feature set 4 contains 3 inputs.

**Figure 9:**
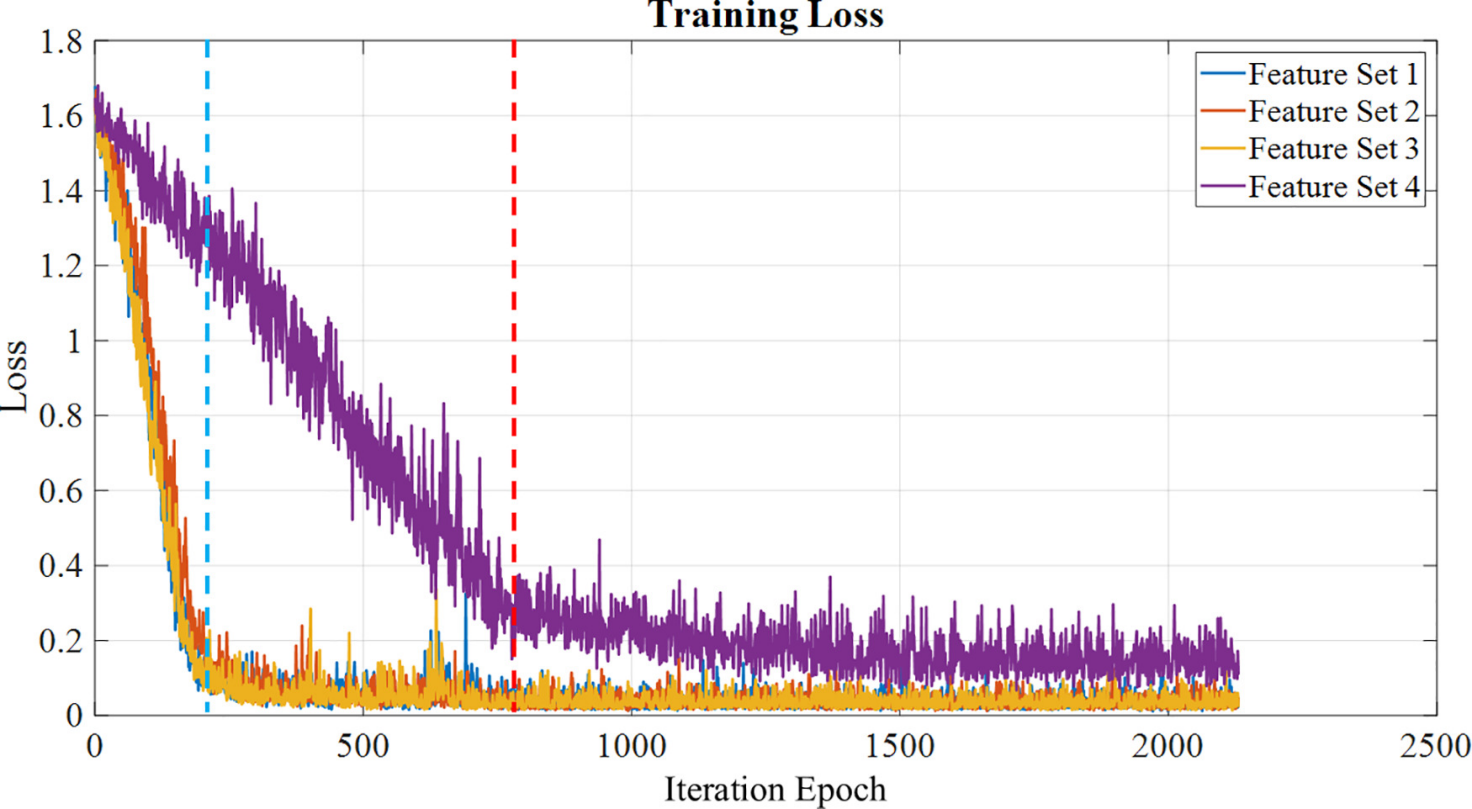
Training loss calculated for each feature set over the epochs, with feature sets 1,2,3 a loss reduction at the 200th iteration (blue line), while feature set 4 shows a loss reduction at the 750th iteration (red line).

The neural network performance during training is evaluated using training loss as a parameter. The number of iterations for the training is 2,130, considering the total sample size is 1,020, the maximum number of epochs is set to be 30, with a mini batch size of 64. Figure 9 shows the training loss for each of the feature sets, showing that the feature sets 1, 2 and 3 tend to learn faster than feature set 4, which has the training loss starting to set at around 750th iteration, while the rest of the feature sets have a steep decrease at around 200th iteration. Once the model is trained, it is evaluated using the test set, and the performance metrics are calculated to visualize the model's classification capability, which is discussed in the following subsection.

### 4.1 Training performance metrics

The performance metrics of the network are accuracy, F1 score, recall, precision, sensitivity, and specificity. The true positive (TP) is the number of correctly predicted labels, and the true negative (TN) is the number of classes that have been predicted wrong. The false positive (FP) is the number of predicted labels that have been predicted to belong to a certain class but do not belong to that class. The false negative is the predicted label which does not belong to the class into which it has been categorized. The accuracy, sensitivity, precision, recall, and F1 score of classification are calculated using equations (5), (6), (7), and (8), respectively. Recall provides information on whether the model is able to identify all the groups in the dataset correctly, while precision provides how precisely the classification occurs using the model.

(5) $$\begin{array}{c} \mathrm{Accuracy}=\frac{TP+TN}{TP+FP+TN+FN} \end{array}$$

(6) $$\mathrm{Precision}=\;\frac{TP}{TP+FP}$$

(7) $$\begin{array}{c} \mathrm{Recall}=\;\frac{TP}{TP+FN} \end{array}$$

(8) $$\begin{array}{c} F1=2\times \frac{\mathrm{Precision}\;\times \mathrm{Recall}}{\mathrm{Precision}+\mathrm{Recall}} \end{array}$$

The macro-averaged performance metrics are listed in Table 4. With the main objective being identifying the best feature set for the classification and identification of tremor progression, it can be seen that the proposed new sensing methodology and parameters work well in accordance with gold standards such as the EMG and MMG_ACC_. It is able to provide a classification accuracy which is competing with that of the remaining sets.

**Table 4:** Averaged performance metrics for the different feature sets for sample size = 1,020.

Feature set	Accuracy (%)	Recall (%)	Precision (%)	F1-score (%)
EMG + MMG_ACC_ + MMG_CSAC_	99	99	99.05	99
EMG + MMG_ACC_	98.04	98.04	98.13	98.04
EMG + MMG_CSAC_	99	99	99.05	99
MMG_ACC_ + MMG_CSAC_	99	99.05	99	99

**Figure 10:**
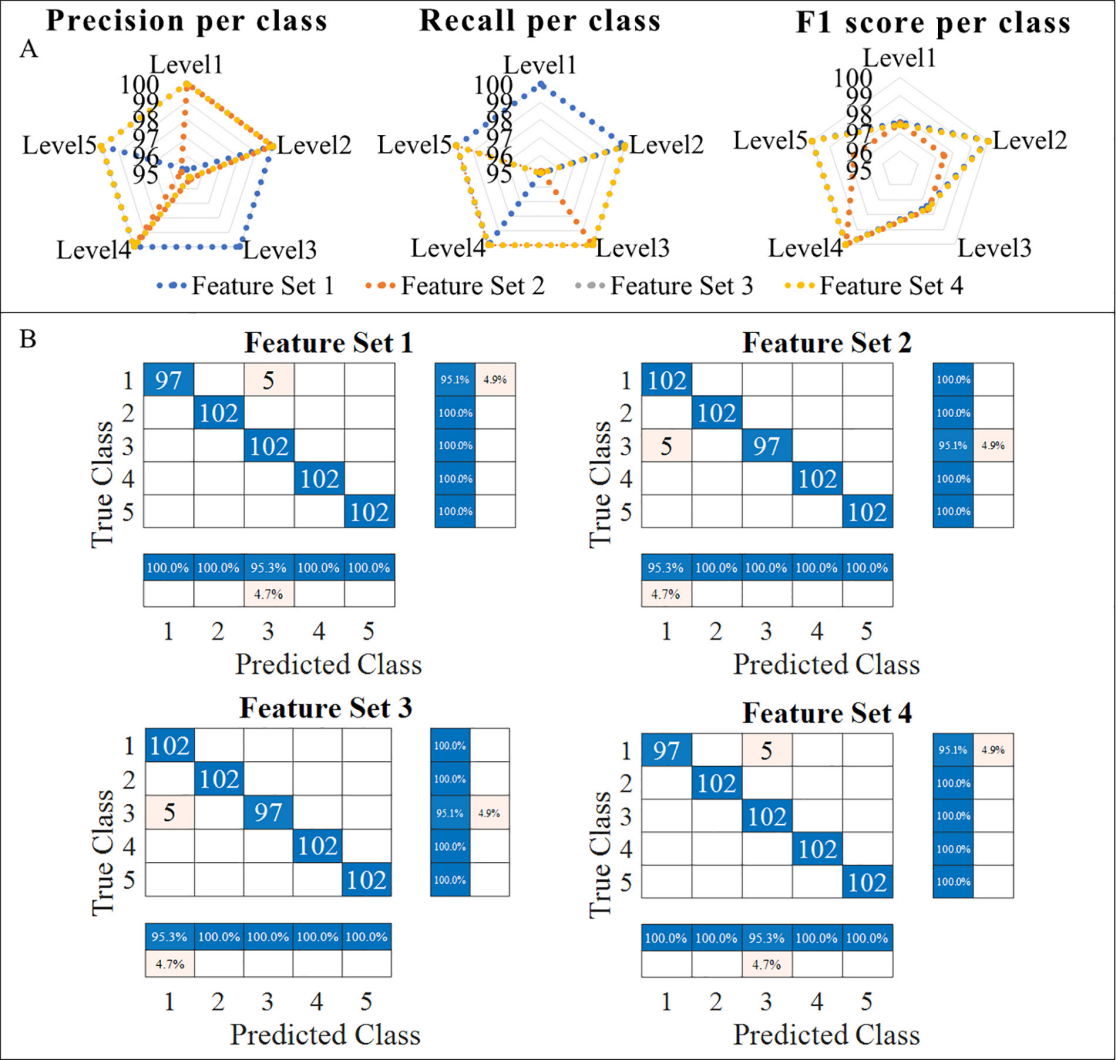
Performance metrics of all four feature sets. **(A)** Precision per class of the feature sets, Recall per class of the feature sets, F1 score per class of the feature sets, **(B)** Confusion matrix for the four feature sets.

On looking at the performance metrics of the feature sets per class as indicated in Figures 10A, it is notable that Feature set 2 has comparatively lesser classification accuracy of 98.04% with respect to the other sets with a classification accuracy of 99%. The confusion chart shown in Figure 10B provides summarised results for true positive, true negative, false positive and false negative with normalized results along row and column. This is also noted in the macro-averaged performance metrics, as seen in Table 4, such as the accuracy and F1 score. The F1 score shows if the classifier can classify both positive and negative sets correctly. The proposed parameter MMG_CSAC_ works on par with the EMG and MMG_ACC,_ as shown by the F1 score of Feature Sets 1, 3, and 4. On looking at Feature Set 1 and Feature Set 4, it can be seen that the precision for Feature Set 4 is less than Feature Set 1 for the Level 3 class. The Level 3 class identifies tremor transition, and it can also be found that the recall of Feature Set 1 is low again in Level 3. From the F1 score, it can be seen that Feature set 4 gives a better classification. At the same time, consideration has to be given to the classification of Level 5 since it is the final tremor achievement level. There are high possibilities for Level 4 and Level 5 to be interchanged, as is the case for Level 3 and Level 2. Hence, more emphasis is laid on the classification of these levels. All the training and performance metrics are provided in the Supplementary Material and Multimedia Link for better understanding.

## 5 Discussion

The main objective of this paper is to present an alternate sensing methodology that can capture the volumetric changes of muscles during movement. The proposed fiducial marker system effectively captures the cross-sectional area changes used to classify the tremor progression stages. The BiLSTM-GRU neural network designed in this work could perform the classification with an accuracy of 99% with the proposed feature set for an augmented dataset of sample size 1,020. The computational complexity reduces since this is a single-channel input feature, unlike EMG, which has multiple channels for assessing multiple muscles. MMG_CSAC_ provides a lumped input of the volumetric changes of all the muscles considered in the extremity chosen. This hybrid network thus aids in efficiently classifying the tremor progression stages using the proposed sensing modality.

In manipulation tasks requiring non-tremulous movements, capturing the origin and transition stages along with quantification of the tremor. This aids in effectively controlling the tremor by implementing filters that will remove the tremor from normal movement or by suppressing mechanisms that can absorb or mitigate the tremor. In robot-assisted surgery, studying and characterizing surgical movements without hindering the surgeon's normal motions is crucial. This can be carried out using the proposed cross-sectional area change feature measured using the simple fiducial markers, which can successfully aid in the classification of the tremulous motions. The drawbacks of the sensor used here are the size of the ArUco marker and the sensitivity of the vision sensor employed. An improvement in these hardware properties would ease the practical implementation of this classification pipeline in real-time in a challenging environment. With respect to the proposed classification pipeline, a cross-validation approach with the test dataset can improve the model. Also, finding the perfect sample size for the training without overfitting the model is quintessential.

## Data Availability

The original contributions presented in the study are included in the article/Supplementary Material, further inquiries can be directed to the corresponding authors.
