# Supplementary material for: Empirical classification of fatigue-induced physiological tremor in robot-assisted manipulation tasks using BiLSTM-GRU network
Source: Front Rehabil Sci. 2025 Jun 17;6:1474203. doi: 10.3389/fresc.2025.1474203 (PMC12231529; doi:10.3389/fresc.2025.1474203)
Supplement: Supplementary file 1 [file Table1.docx]

Supplementary Material

# RESULTS FOR SAMPLE SIZE: 1020

## Performance metrics

Table 1. Precision per class

| Precision | Level1 | Level2 | Level3 | Level4 | Level5 |
| --- | --- | --- | --- | --- | --- |
| Feature Set 1 | 95.24 | 100 | 100 | 100 | 100 |
| Feature Set 2 | 100 | 100 | 95.33 | 100 | 95.33 |
| Feature Set 3 | 100 | 100 | 95.24 | 100 | 100 |
| Feature Set 4 | 100 | 100 | 95.24 | 100 | 100 |

Table 2. Recall per class

| Recall | Level1 | Level2 | Level3 | Level4 | Level5 |
| --- | --- | --- | --- | --- | --- |
| Feature Set 1 | 100 | 100 | 95 | 100 | 100 |
| Feature Set 2 | 95.1 | 95.1 | 100 | 100 | 100 |
| Feature Set 3 | 95 | 100 | 100 | 100 | 100 |
| Feature Set 4 | 95 | 100 | 100 | 100 | 100 |

Table 3. F1 score per class

| F1 Score | Level1 | Level2 | Level3 | Level4 | Level5 |
| --- | --- | --- | --- | --- | --- |
| Feature Set 1 | 97.56 | 100 | 97.44 | 100 | 100 |
| Feature Set 2 | 97.49 | 97.49 | 97.61 | 100 | 97.61 |
| Feature Set 3 | 97.44 | 100 | 97.56 | 100 | 100 |
| Feature Set 4 | 97.44 | 100 | 97.56 | 100 | 100 |

## Training plots

### Feature Set 1


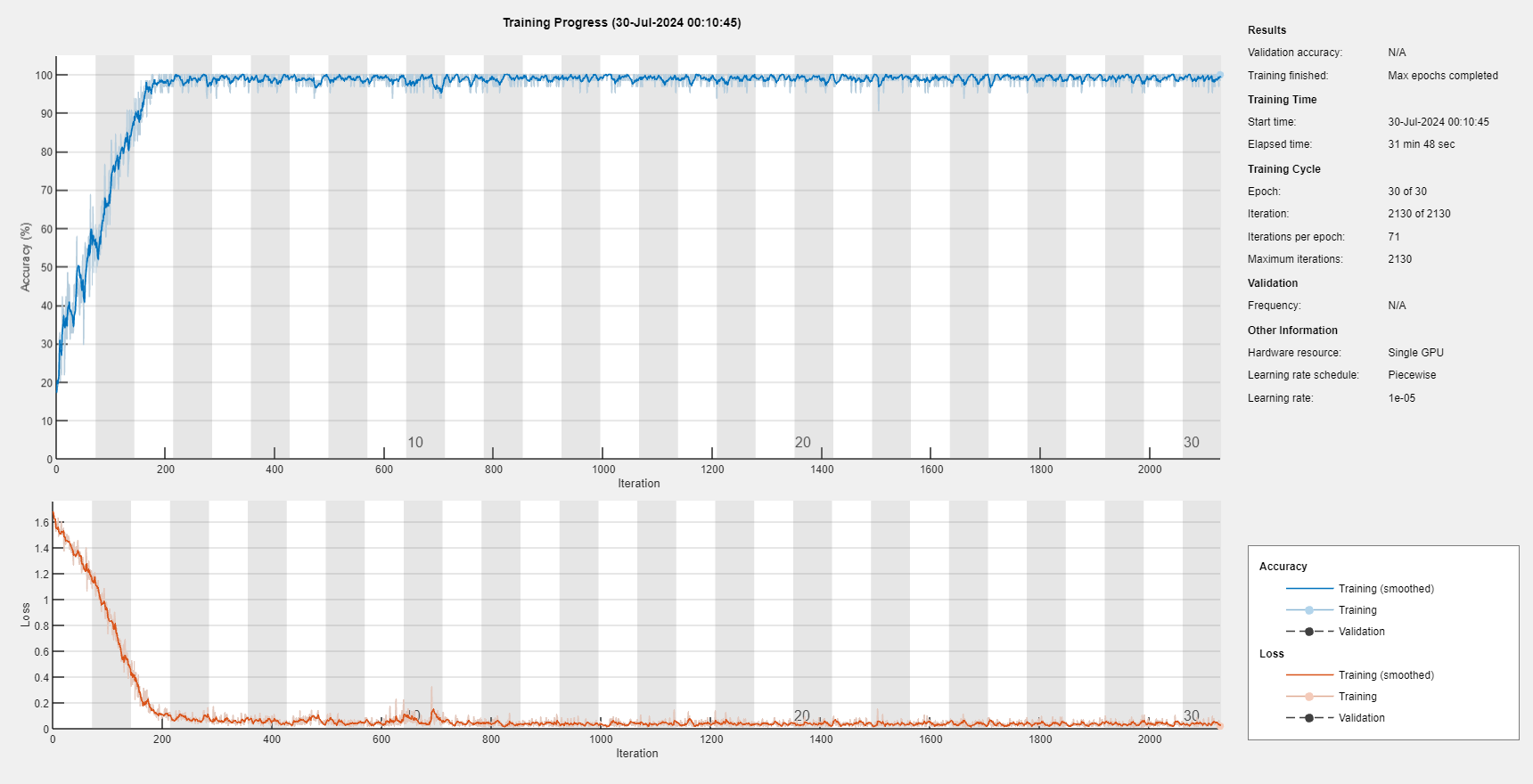


Figure 1. Training plot generated from MATLAB for Feature Set 1


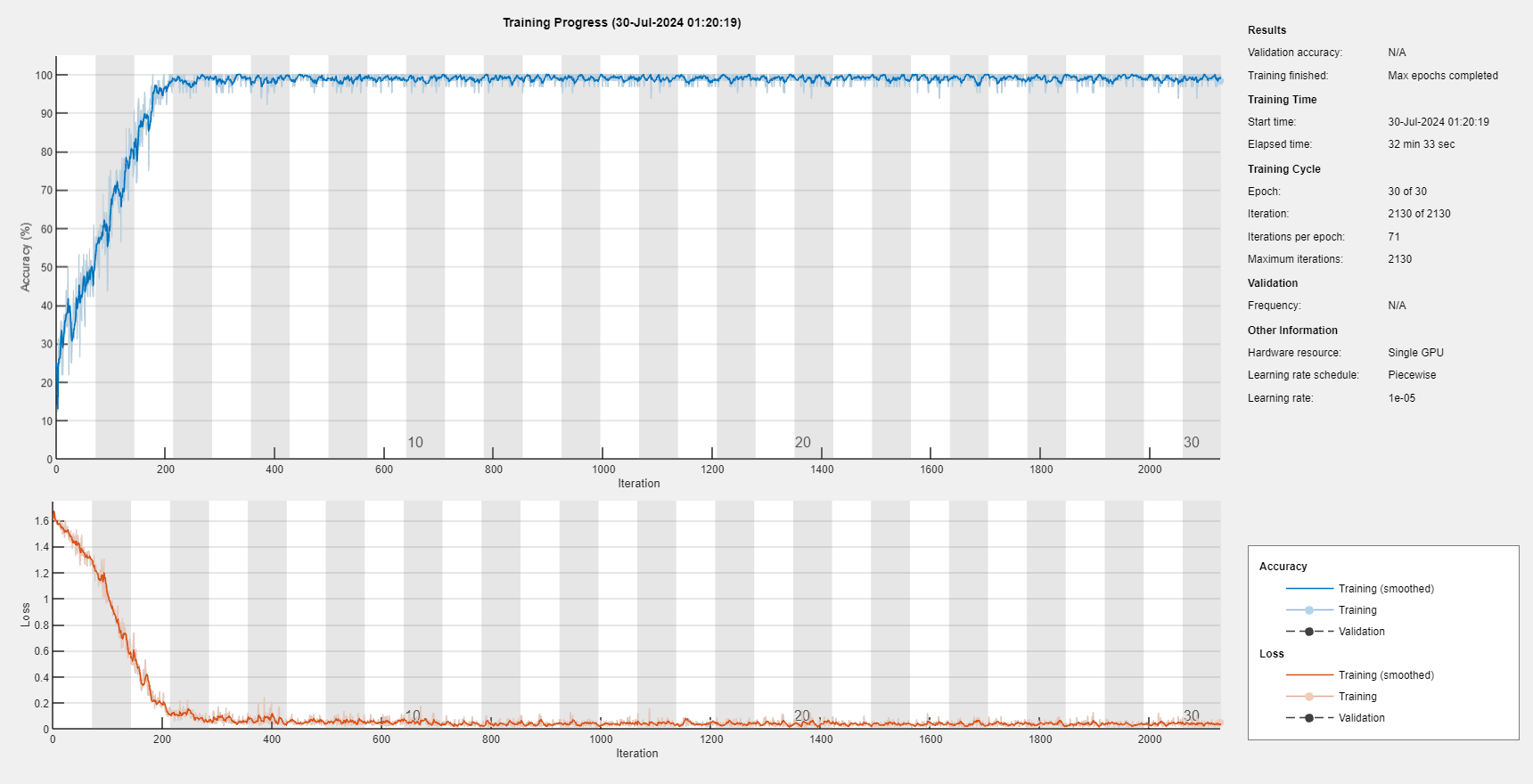


Figure 2. Training plot generated from MATLAB for Feature Set 2


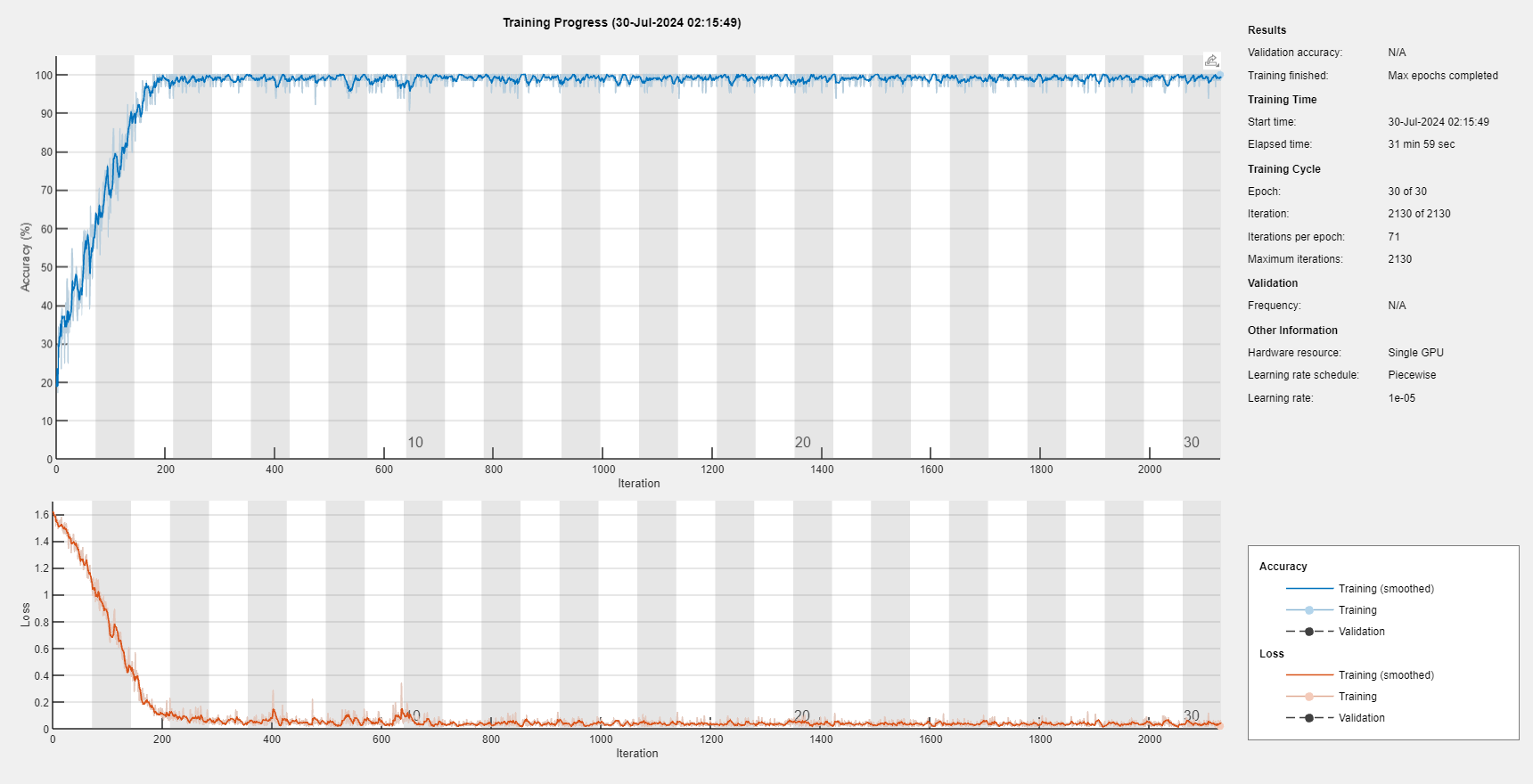


Figure 3. Training plot generated from MATLAB for Feature Set 3


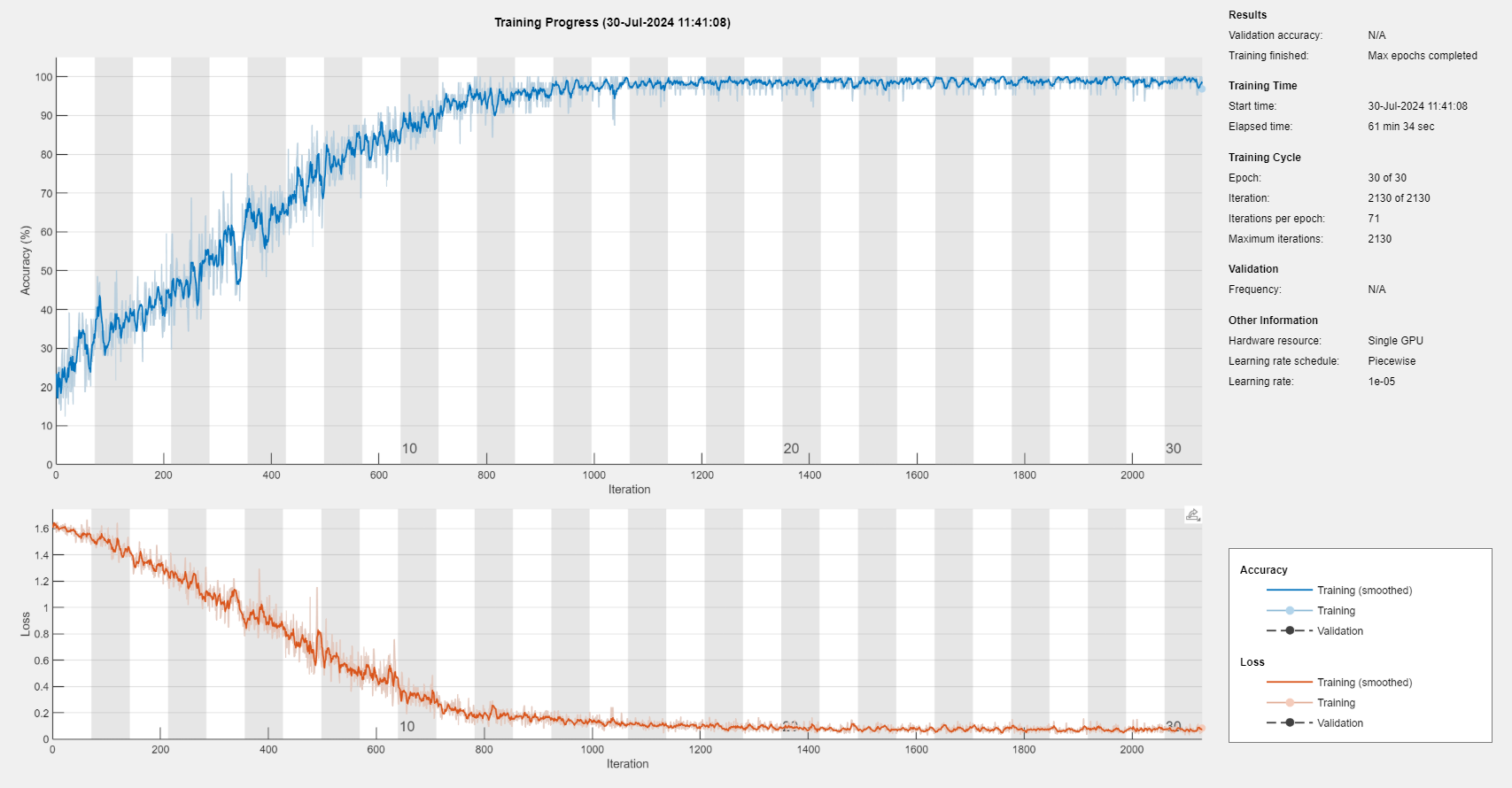


Figure 4. Training plot generated from MATLAB for Feature Set 4

# RESULTS FOR SAMPLE SIZE: 1020

## Performance metrics

Table 3. Precision per class

| Precision | Level1 | Level2 | Level3 | Level4 | Level5 |
| --- | --- | --- | --- | --- | --- |
| Feature Set 1 | 60 | 57.14 | 53.85 | 75 | 71.43 |
| Feature Set 2 | 50 | 60 | 56.25 | 66.67 | 83.33 |
| Feature Set 3 | 64.28 | 40 | 61.5 | 55.55 | 55.55 |
| Feature Set 4 | 25 | 28.57 | 33.33 | 20 | 44.44 |

Table 4. Recall per class

| Recall per class: | Level1 | Level2 | Level3 | Level4 | Level5 |
| --- | --- | --- | --- | --- | --- |
| Feature Set 1 | 90 | 40 | 70 | 60 | 50 |
| Feature Set 2 | 70 | 30 | 90 | 60 | 50 |
| Feature Set 3 | 90 | 20 | 80 | 50 | 50 |
| Feature Set 4 | 30 | 20 | 40 | 20 | 40 |

Table 5. F1 score per class

| F1 Score per class | Level1 | Level2 | Level3 | Level4 | Level5 |
| --- | --- | --- | --- | --- | --- |
| Feature Set 1 | 72 | 47.06 | 60.87 | 66.67 | 58.82 |
| Feature Set 2 | 58.33 | 40 | 69.3 | 63.15 | 62.5 |
| Feature Set 3 | 75 | 26.67 | 69.56 | 52.63 | 52.63 |
| Feature Set 4 | 27.27 | 23.52 | 36.36 | 20 | 42.1 |


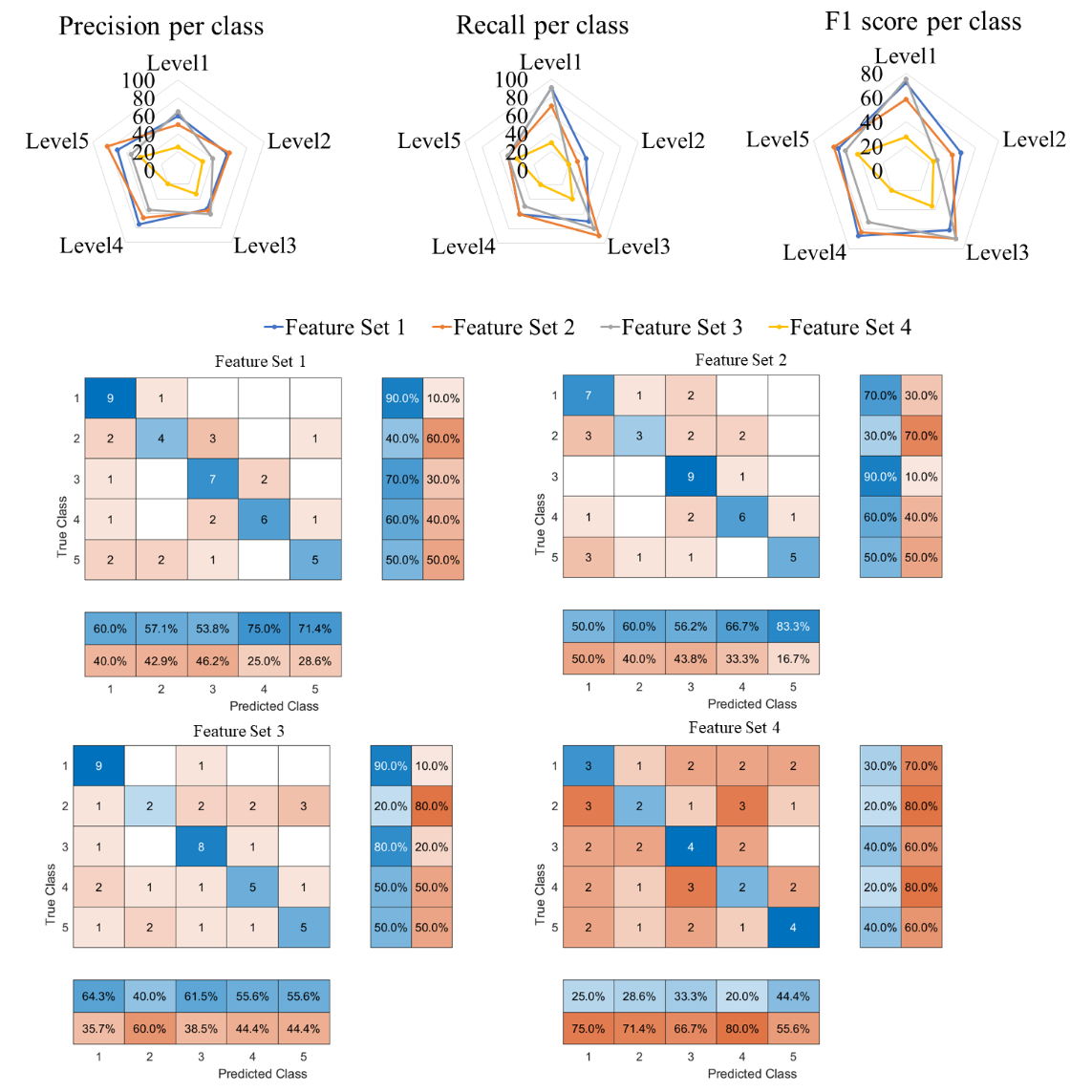


Figure 5. Graphical representation of the performance metrics of Sample Size = 100

## Confusion chart for the sample size = 100


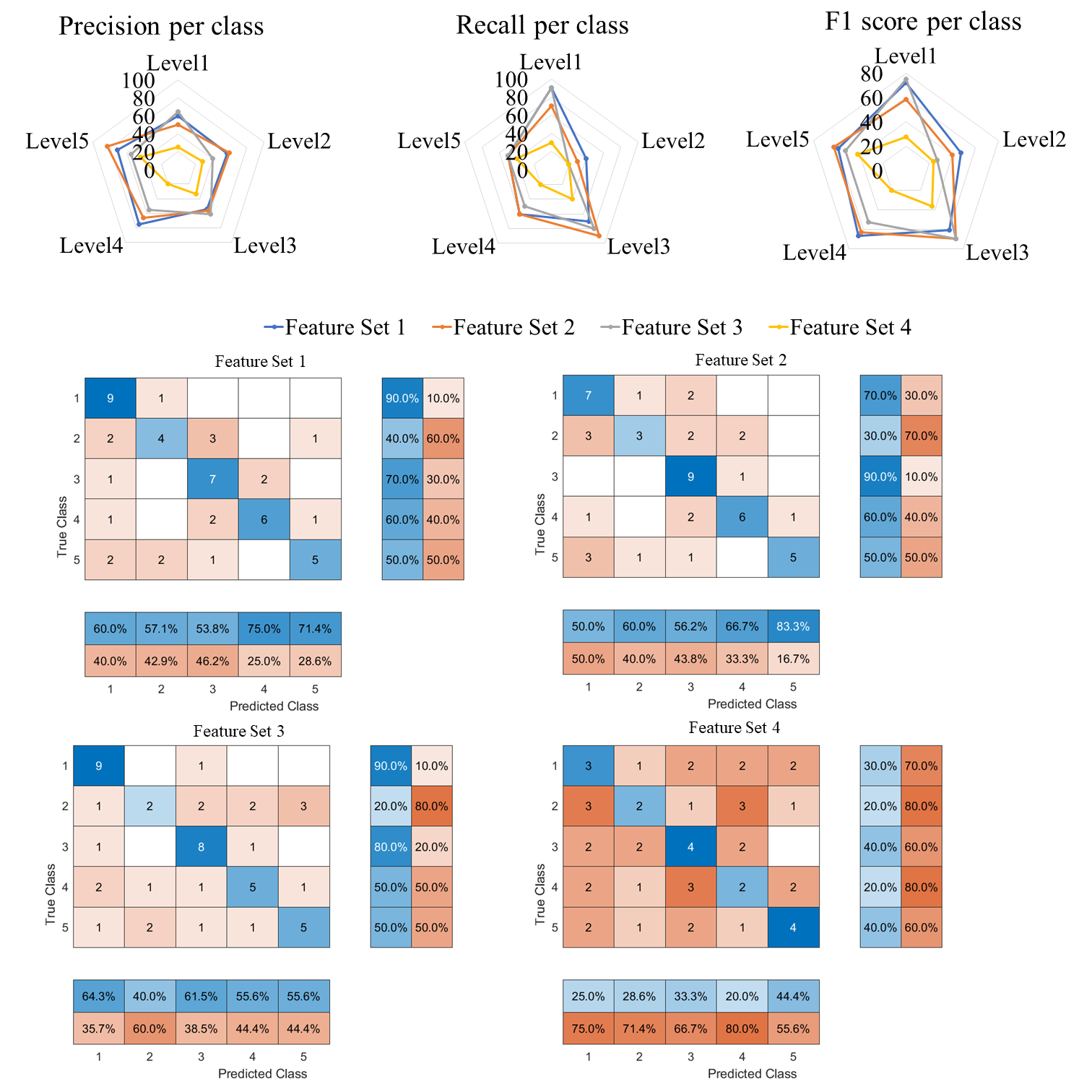


Figure 6. Confusion matrix of features sets 1,2,3 and 4

## Training plots generated from MATLAB during model training


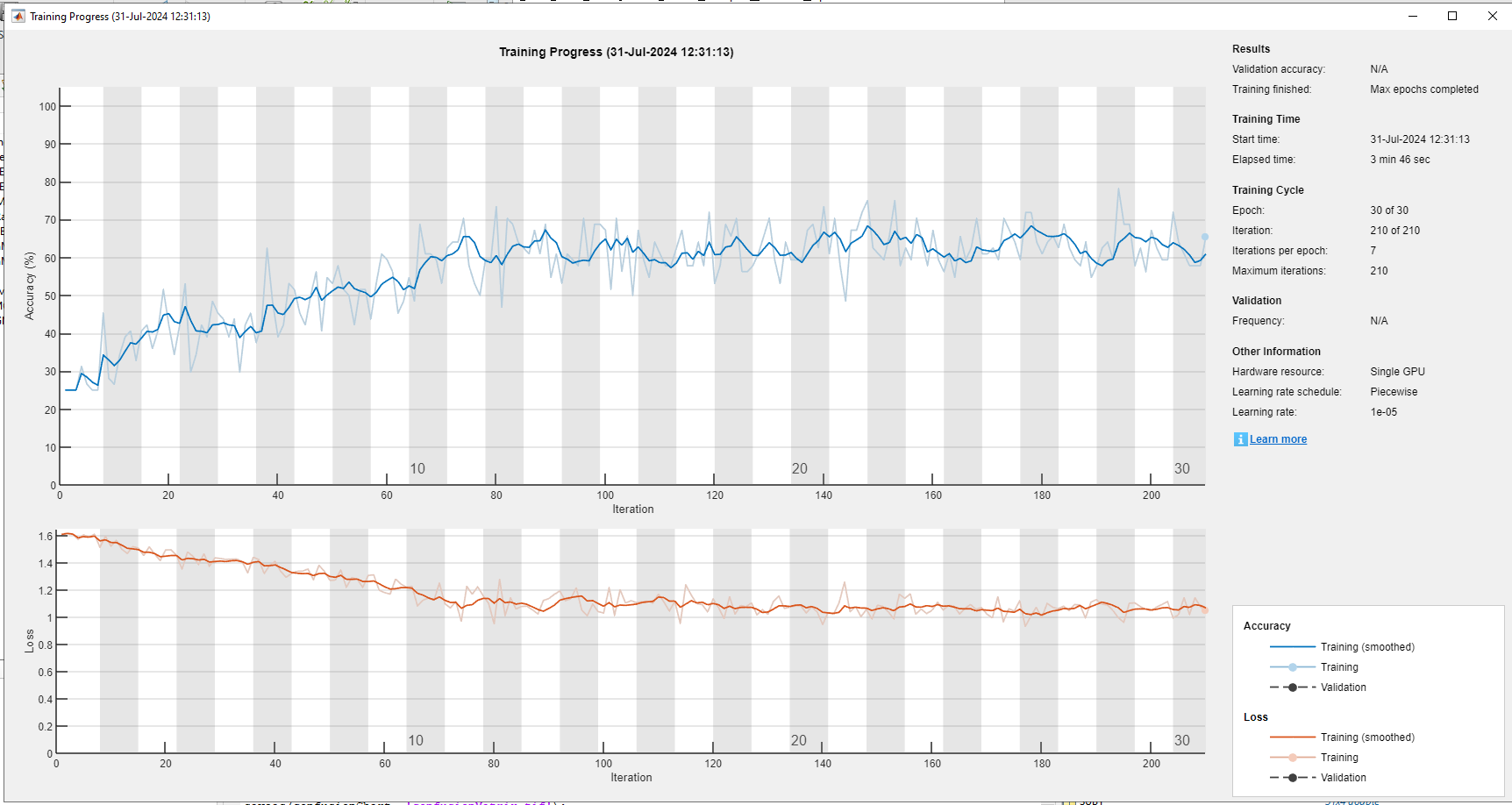


Figure 7. Training plot generated from MATLAB for Feature Set 1


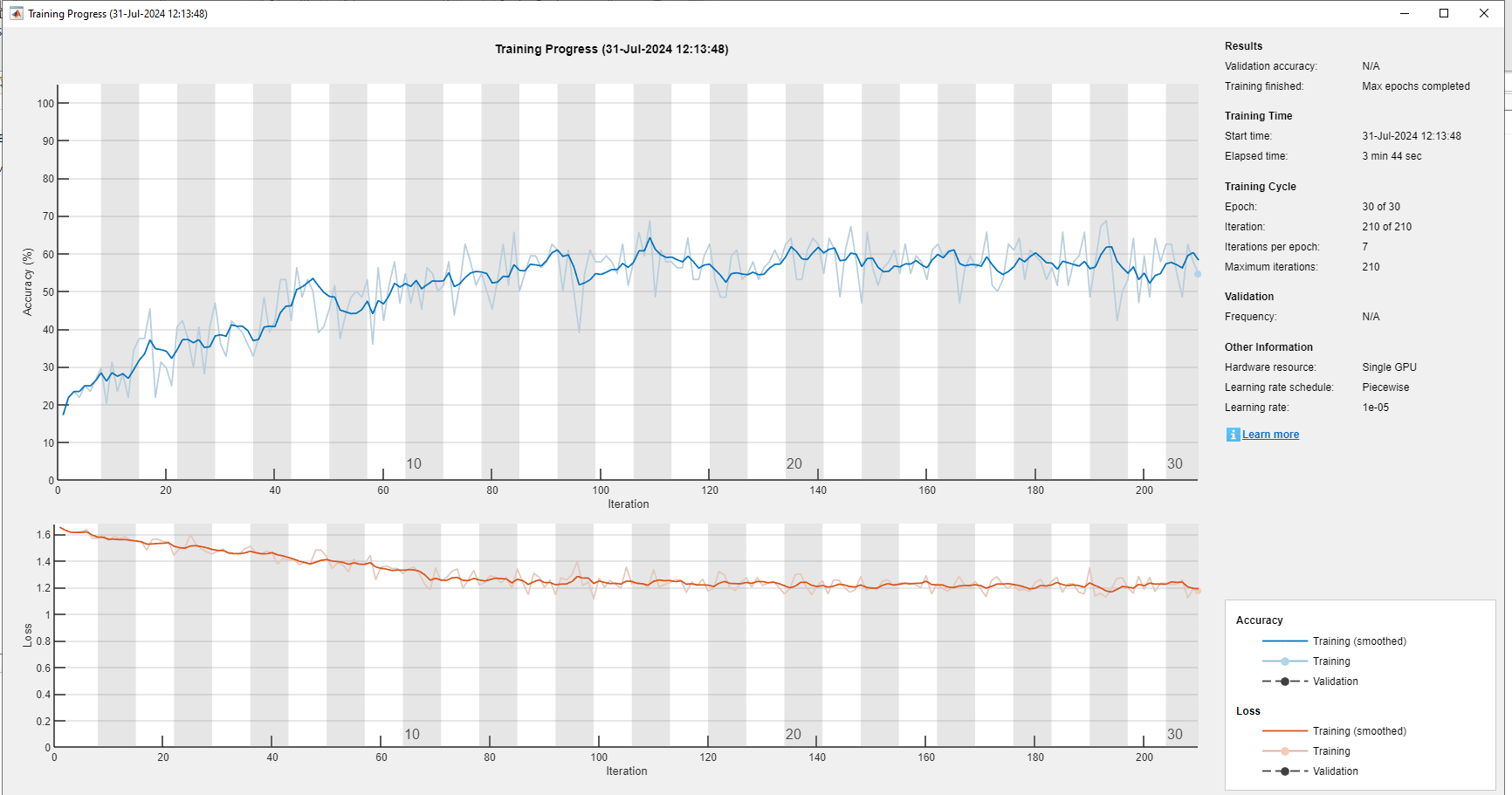


Figure 8. Training plot generated from MATLAB for Feature Set 2


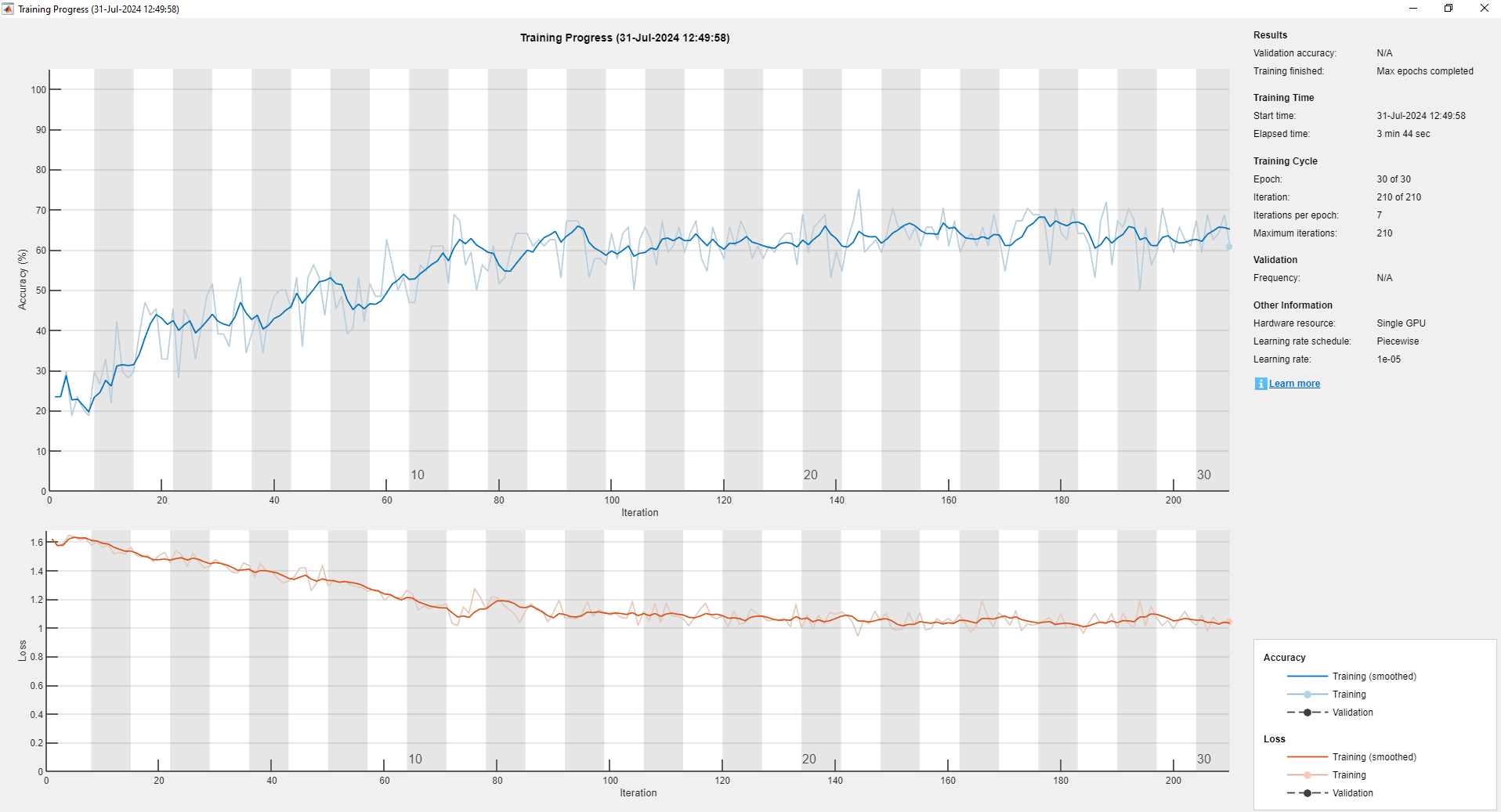


Figure 9. Training plot generated from MATLAB for Feature Set 3


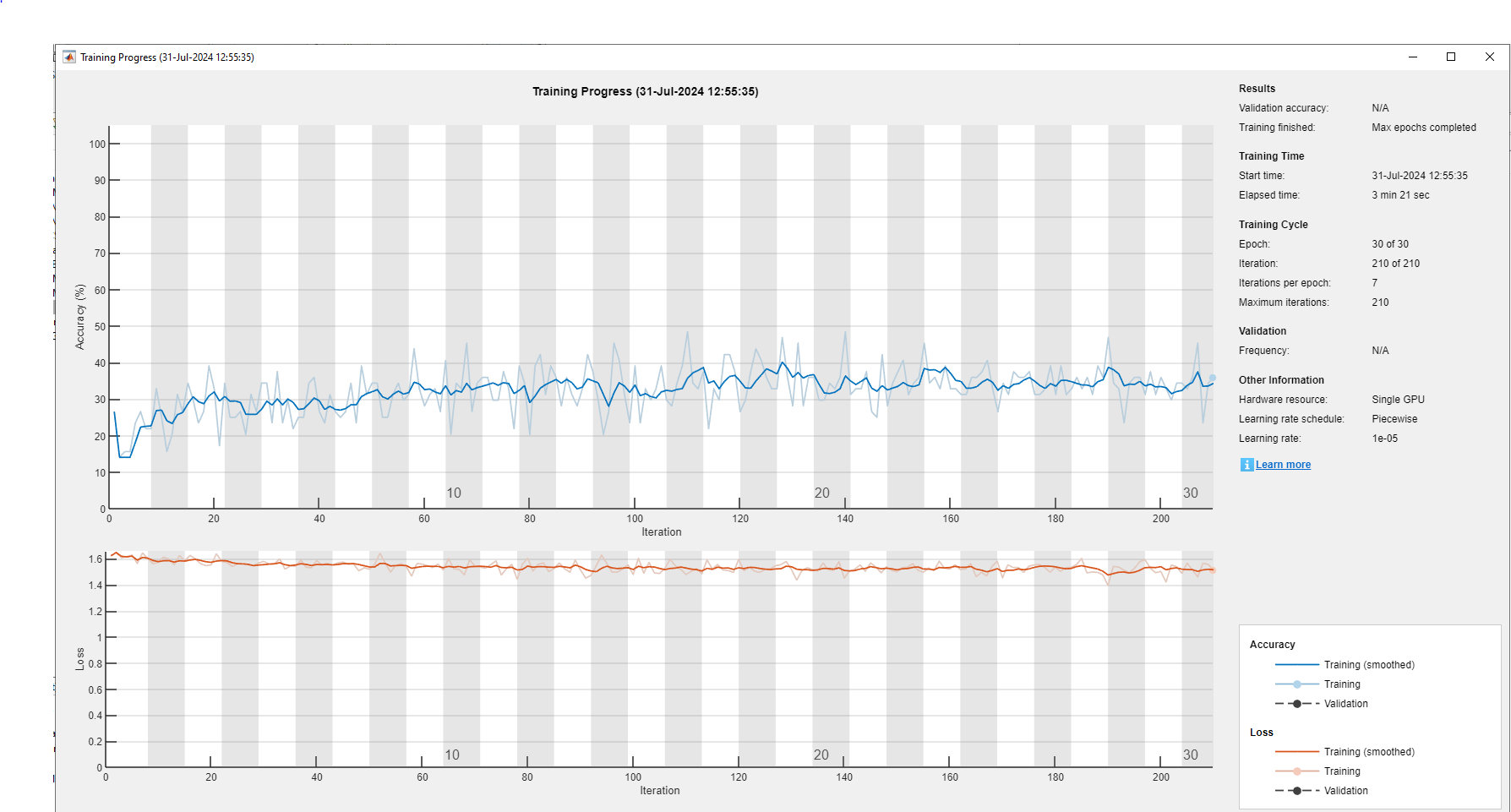


Figure 10. Training plot generated from MATLAB for Feature Set 4

Multimedia Link: For better understanding, a [multimedia link](https://youtu.be/Jqwb4Pm65I4) is provided which explains the proposed methodology in detail.
